# The DEC2‐SCN2A Axis is Essential for the Anticonvulsant Effects of Cannabidiol by Modulating Neuronal Plasticity

**DOI:** 10.1002/advs.202416315

**Published:** 2025-07-11

**Authors:** Huifang Song, Yifan Wang, Lili Wang, Chang Guo, Shiqi Liu, Yi Rong, Jiawen Tian, Chao Peng, Yuying Shao, Zhixiong Ma, Na Li, Jingliang Zhang, Zijun Peng, Xu Yan, Hangwei Fa, Xinyue Ma, Jie Dong, Jinping Ji, Chen Yang, Haocheng Chen, Jing Liang, Qi Sun, Yang Yang, Weining Ma, Zhuo Huang

**Affiliations:** ^1^ State Key Laboratory of Natural and Biomimetic Drugs Department of Molecular and Cellular Pharmacology School of Pharmaceutical Sciences Peking University Beijing 100191 China; ^2^ Institute of Genetics and Developmental Biology Chinese Academy of Sciences Beijing 100101 China; ^3^ Borch Department of Medicinal Chemistry and Molecular Pharmacology College of Pharmacy & Purdue Institute for Integrative Neuroscience Purdue University West Lafayette IN 47907 USA; ^4^ Department of Biochemistry and Molecular Biology School of Basic Medical Sciences Peking University Beijing 100191 China; ^5^ Department of Neurosurgery Shengjing Hospital of China Medical University Shenyang 110022 China; ^6^ IDG/McGovern Institute for Brain Research Peking University Beijing 100871 China; ^7^ Ningbo Institute of Marine Medicine Peking University Ningbo 315832 China

**Keywords:** cannabidiol, DEC2, epilepsy, intrinsic plasticity, NaV1.2 channel, synaptic plasticity, transcription regulation

## Abstract

Impairment of neuronal plasticity is involved in a spectrum of neurological disorders such as epilepsy, yet its regulatory mechanisms remain incompletely understood. Here, it is reported that the basic helix‐loop‐helix transcription factor DEC2 serves as a pivotal regulator of both neuronal plasticity and epileptogenesis through its repression of sodium voltage‐gated channel alpha subunit 2 (SCN2A). Knockdown of DEC2 in hippocampal neurons elevates intrinsic excitability and synaptic transmission, exacerbating seizure susceptibility and severity. Conversely, overexpression of DEC2 in hippocampus reduces intrinsic excitability and synaptic transmission, ultimately decreasing seizure susceptibility. Mechanistically, DEC2 functions as a transcriptional repressor of *Scn2a* by directly binding class B E‐boxes (CACGTG) in its promoter. Additionally, DEC2 forms complexes with myoblast determination protein 1 (MYOD1) and occupies the CAGCTG E‐boxes within the *Scn2a* promoter; however, this interaction does not affect *Scn2a* transcription in vivo. These findings also reveal that cannabidiol (CBD) can modulate the DEC2‐SCN2A axis. Notably, CBD predominantly enhances DEC2's direct transcriptional repression of SCN2A. In summary, this study identifies DEC2 as a critical regulator of neuronal plasticity in epilepsy progression, suggesting a novel therapeutic pathway for epilepsy treatment.

## Graphical abstract

This study identifies DEC2 as a critical transcriptional repressor of *Scn2a* that directly binds E‐box motifs in its promoter to regulate neuronal plasticity and epileptogenesis. Functional manipulation of DEC2 alters seizure susceptibility in vivo. Cannabidiol enhances this repression, unveiling a novel DEC2‐SCN2A axis underlying its anticonvulsant action and proposing DEC2 as a promising target for epilepsy intervention.

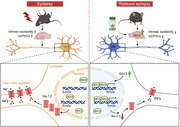

## 1 Introduction

Activity‐dependent neuronal plasticity, the ability of the nervous system to change its function in response to prior experience, plays a fundamental role in the establishment and refinement of neural networks during learning and memory. Intrinsic and synaptic plasticity represent the two most common plasticity mechanisms.^[^ ^1^ ^]^ Intrinsic plasticity involves alterations in local or cell‐wide neuronal excitability, influencing the likelihood of a neuron firing an action potential in response to excitatory synaptic inputs.^[^ ^2^ ^]^ Synaptic plasticity refers to the modification of the strength or efficacy of synaptic transmission between neurons.^[^ ^3^ ^]^ The impairment of neuronal plasticity has been linked to various neurological and psychiatric disorders.^[^ ^4^ ^]^ However, the precise regulatory mechanisms are still poorly understood.

Secondary epilepsy, also referred to as symptomatic epilepsy, is frequently induced by cerebral injuries, including those resulting from trauma or infection.^[^ ^5^ ^]^ Subsequent to the initial insult, the brain undergoes a complex cascade of neuronal plasticity alterations, encompassing molecular, cellular, and network‐level reorganization. This dynamic remodeling ultimately leads to the development of a chronic epileptic state.^[^ ^6^ ^]^ Central to these alterations are transcription factors, which play a pivotal role in regulating plasticity‐related gene expression. The participation of these transcription factors in the pathogenesis of epilepsy has been documented across numerous studies.^[^ ^6^, ^7^ ^]^ However, the mechanisms through which the brain undergoes plastic changes following the injury are still not fully understood, and the role of transcriptional regulation in this process remains elusive.

Ion channels scaffolded within neurons are fundamental to the modulation of neuronal plasticity. Among the ion channels that influence neuronal plasticity, voltage‐gated sodium channels, particularly Na_V_1.2 encoded by the *Scn2a* gene, play a significant role. In immature neurons, Na_V_1.2 is thought to control action potential (AP) initiation and modulate early developmental axonal excitability.^[^ ^8^ ^]^ In mature neurons, Na_V_1.2 is involved in facilitating the backpropagation of APs from the axon initial segment (AIS) to the soma and dendrites.^[^ ^8^, ^9^ ^]^ These backpropagating APs can influence various functions, including dendritic excitability, activity‐dependent gene transcription, synaptic integration, and synaptic plasticity.^[^ ^8^, ^10^ ^]^ Therefore, deciphering the transcriptional regulation of SCN2A is critical for unraveling the molecular architecture underlying neuronal plasticity.

Cannabidiol (CBD), a non‐psychoactive phytocannabinoid derived from Cannabis sativa, has emerged as a promising therapeutic agent for drug‐resistant epilepsies. Accumulating evidence indicates that CBD can modulate neuronal excitability through multiple mechanisms, including inhibiting T‐type calcium channels and voltage‐gated sodium channels,^[^ ^11^ ^]^ which collectively suppress pathological hyperexcitability in epileptic circuits. However, the precise molecular targets and mechanisms underlying seizure control remain elusive, particularly the long‐term transcriptional regulation induced by CBD treatment. Addressing these unresolved issues is essential for advancing the optimization of CBD‐based therapies and will provide valuable directions for investigating novel epigenetic and transcriptional pathways in epilepsy treatment.

In this study, we employed weighted gene co‐expression network analysis (WGCNA) to identify the basic helix‐loop‐helix (bHLH) family member e41 (BHLHE41, also known as DEC2) as a critical regulator of intrinsic and synaptic neuronal plasticity in temporal lobe epilepsy (TLE). DEC2 is upregulated in response to epileptic seizures, and represses the transcription of Na_V_1.2 by binding to the *Scn2a* gene promoter, thereby mitigating aberrant neuronal excitability and seizure susceptibility. Furthermore, our research highlights the DEC2‐SCN2A axis as a crucial molecular pathway through which CBD mediates its anticonvulsant effects in epilepsy. We demonstrate that CBD upregulates DEC2, both in vitro and in vivo, which in turn downregulates SCN2A, with DEC2 pre‐knockdown abolishing these effects. Collectively, our findings provide a novel regulatory axis linking neuronal activity to plasticity and suggest that transcriptional regulation plays a crucial role in modulating behavioral changes during both physiological and pathological brain processes.

## 2 Results

### 2.1 DEC2 is Upregulated in Rodent and Human Epileptic Tissues

**Figure 1:**
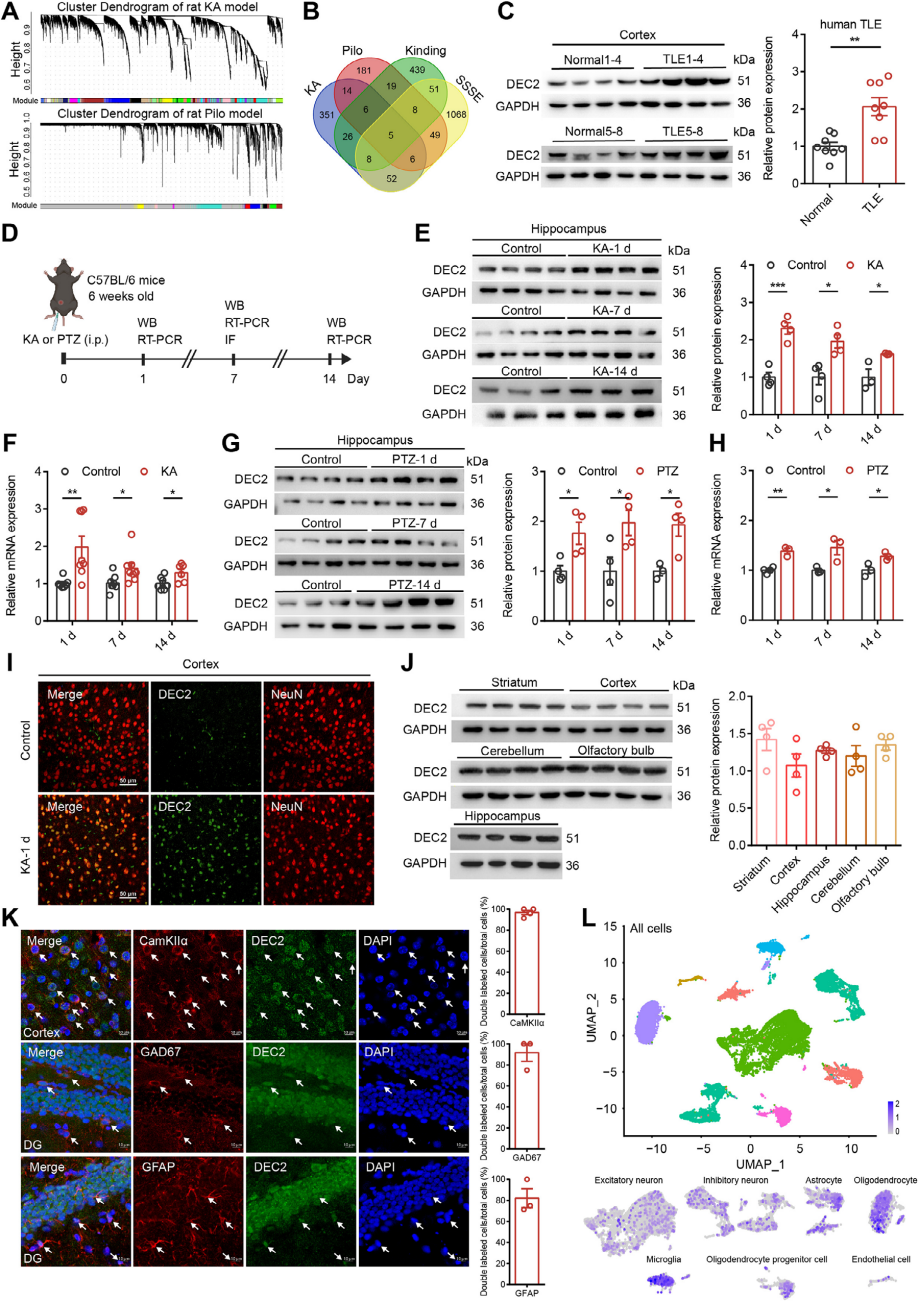
DEC2 is upregulated in rodent and human epileptic tissues. A) Co‐expression network analyses of rat KA and Pilo models. The gene modules identified by WGCNA are shown by cluster dendrogram in which the branches correspond to modules and each leaf in the branch represents one probe. B) Venn diagram showing the overlaps of genes identified in the MEred of rat KA model, MEblue of rat Pilo model, MEgreen of rat kindling model, and MEbrown of rat SSSE model. C) Western blot and quantification showing DEC2 expression in the temporal lobe tissues from normal human or TLE patients. n = 8, **p < 0.01, unpaired two‐tailed Student's t‐test. D) Schematic diagram of experimental design for seizure model induction and tissue sampling in mice. E) Western blot and quantification showing DEC2 expression in the hippocampus at 1‐, 7‐, and 14‐ days after status epilepticus in mice KA model. n = 4 for 1 d and 7 d, n = 3 for 14 d, *p < 0.05, ***p < 0.001, two‐way ANOVA with Bonferroni's multiple‐comparisons test. F) The mRNA levels of *Dec2* in the hippocampus were assessed by RT‐PCR at 1‐, 7‐, and 14‐ days after status epilepticus in mice KA model. The levels of mRNA were normalized to that of β‐actin. n = 6 to 8 for each group, *p < 0.05, ***p < 0.001, two‐way ANOVA with Bonferroni's multiple‐comparisons test. G) Western blot and quantification showing DEC2 expression in the hippocampus at 1‐, 7‐, and 14‐ days after acute seizure in mice PTZ model. n = 3 to 4 for each group, *p < 0.05, two‐way ANOVA with Bonferroni's multiple‐comparisons test. H) The mRNA levels of *Dec2* in the hippocampus were assessed by RT‐PCR at 1‐, 7‐, and 14‐ days after acute seizure in mice PTZ model. The levels of mRNA were normalized to that of β‐actin. n = 3 to 4 for each group, *p < 0.05, **p < 0.01, two‐way ANOVA with Bonferroni's multiple‐comparisons test. I) Immunofluorescence staining of DEC2 protein (green) in mouse cortex 7 days post‐KA injection. Scale bar = 50 µm. J) Western blot and quantification of DEC2 in mice different brain regions. n = 4 per group. K) Representative images of DEC2 (green) with CamKIIα (red), GAD67 (red), and GFAP (red) immunostaining in mouse brain. White arrows mark co‐localized puncta. Right panels show the percentage of co‐localized cells among total marker‐positive cells. Scale bar = 10 µm. L) (Upper) UMAP representation of all cell types from the hippocampus of mouse. (Bottom) Distribution of *Dec2* transcripts among subsets of cells. The color spectrum from gray to purple indicates expression levels from low to high. Data were represented as mean ± SEM.

To unravel the molecular basis underlying epilepsy, we conducted a comprehensive study utilizing the powerful tool of WGCNA.^[^ ^12^ ^]^ This approach enabled us to identify key modules (highly co‐expressed clusters of genes) and critical genes associated with epilepsy. The weighted gene co‐expression network was constructed using the transcriptome data of GSE47752 obtained from the Gene Expression Omnibus (GEO), which contained four rat models of status epilepticus (SE): kainic acid (KA), pilocarpine (Pilo), kindling, and self‐sustained SE (SSSE) (**Figure** **1**A). After removing genes with variability or missing values, we constructed the co‐expression matrix for each rat model. The WGCNA analysis yielded 25 modules in the KA model, 10 in the Pilo model, 6 in the kindling model, and 30 in the SSSE model, each represented by a unique color. These gene modules were visualized through a cluster dendrogram. Notably, among the identified modules, the red module (MEred) in the KA model (Pearson correlation coefficient = 0.47, *p* = 0.02), the blue module (MEblue) in the Pilo model (Pearson correlation coefficient = 0.5, *p* = 1e−04), the green module (MEgreen) in the kindling model (Pearson correlation coefficient = 0.3, *p* = 0.04), and the brown module (MEbrown) in the SSSE model (Pearson correlation coefficient = 0.36, *p* = 0.1) exhibited the strongest correlations with the epilepsy phenotype. To identify critical genes within these modules, we generated a scatter plot correlating gene significance (GS) with module membership (MM). Genes located in the upper right corner of the scatter plots were considered candidate critical genes due to their strong association with the phenotype (high GS) and their central role within the module (high MM) (Figure S1A–D, Supporting Information). Across all four datasets, only five genes were consistently present in the MEred, MEblue, MEgreen, and MEbrown modules. Among these, *Dec2* (also known as *Bhlhe41*, *Bhlhb3*, *Sharp1*) distinguished as the most prominent candidate gene (Figure 1B; Figure S1E, Supporting Information). Transcription factor DEC2 belongs to the bHLH superfamily. It displays a fairly ubiquitous expression pattern but is highly abundant in skeletal muscle and brain.^[^ ^13^ ^]^ Within the brain, DEC2 has predominantly been investigated for its role in circadian rhythms and sleep regulation,^[^ ^14^ ^]^ while its other potential functions remain largely unexplored. A recent pivotal study also reports a substantial upregulation of DEC2 in the hippocampal region of mouse subjected to experimental TLE.^[^ ^15^ ^]^ However, the molecular mechanisms underlying this upregulation of DEC2 in epileptic tissues remain entirely unknown, which positions DEC2 as an intriguing target for further investigation in the field of TLE.

To investigate the potential involvement of DEC2 in TLE, we examined the expression of DEC2 in temporal lobe tissues surgically resected from patients. Our findings revealed a notable enhancement in DEC2 expression within TLE samples when compared to adjacent non‐epileptic tissue controls (Figure 1C; and Table S1, Supporting Information). We also referred to the latest updates in human GEO datasets and validated enhanced *Dec2* mRNA level across three separate datasets related to human epilepsy, including those for mesial TLE from GSE186334, focal cortical dysplasia (FCD) from GSE128300, and FCD type IIb from GSE213488 (Figure S1F–H, Supporting Information). Typically, FCD constitutes a major cause of pharmacoresistant epilepsy and is often accompanied by the onset of epileptic seizures.^[^ ^16^ ^]^ To further investigate the alterations of DEC2 in epileptogenesis, we employed two generalized seizure models in mice induced by KA and pentylenetetrazol (PTZ), respectively (Figure 1D). Intraperitoneal administration of KA or PTZ in mice led to an increase in DEC2 at both the protein and mRNA levels 1‐, 7‐, and 14‐ days post‐treatment, as evidenced by western blot and quantitative real‐time reverse transcription (RT)‐PCR assay (Figure 1E–H). We subsequently confirmed the upregulation of DEC2 in the temporal cortex at 7‐day post‐kainic acid injection by performing immunofluorescence staining (Figure 1I). Together, these results indicate that the expression of DEC2 is elevated in both rodent and human epileptic tissues, implying a potential involvement of DEC2 in the pathogenesis and progression of epilepsy.

Previous studies have highlighted the roles of DEC2 in certain brain regions, yet the pattern of expression and localization of DEC2 is not fully understood. To elucidate the potential contributions of DEC2 to normal neuronal function, we initially assessed the expression of DEC2 in the mouse brain using western blot. The results revealed that DEC2 was ubiquitously expressed throughout the mouse brain, including the striatum, cortex, hippocampus, cerebellum, and olfactory bulb (Figure 1J). Immunofluorescence staining of sections from wild‐type mice showed that DEC2 was predominantly localized within neuronal nuclei, as demonstrated by DEC2‐positive cells that typically co‐localize with the neuronal nuclei marker NeuN in the cortex, hippocampal CA1 and dentate gyrus (DG) regions (Figure S1I, Supporting Information). Subsequently, we investigated the cell‐type‐specific expression pattern of DEC2. To determine which type of cell expresses DEC2, we stained brain slices with cell‐type specific markers. DEC2 was found to co‐localize not only with the excitatory neuronal marker CamKIIα and the GABAergic neuronal marker glutamic acid decarboxylase 67 (GAD67), but also with the astrocytic marker glial fibrillary acidic protein (GFAP) (Figure 1K). This indicates that DEC2 is expressed in both excitatory glutamatergic and inhibitory GABAergic neurons, as well as in astrocytes. To further validate the cellular expression pattern of DEC2, we consulted the publicly available mouse single‐cell RNA sequencing database GSE190453,^[^ ^17^ ^]^ which revealed that DEC2 is indeed widely expressed across diverse cell subtypes within the brain (Figure 1L). Collectively, these results suggest that DEC2 may play a broad and pivotal role in the maintenance of normal brain function.

### 2.2 DEC2 Suppresses Seizures Susceptibility and Epileptogenesis

**Figure 2:**
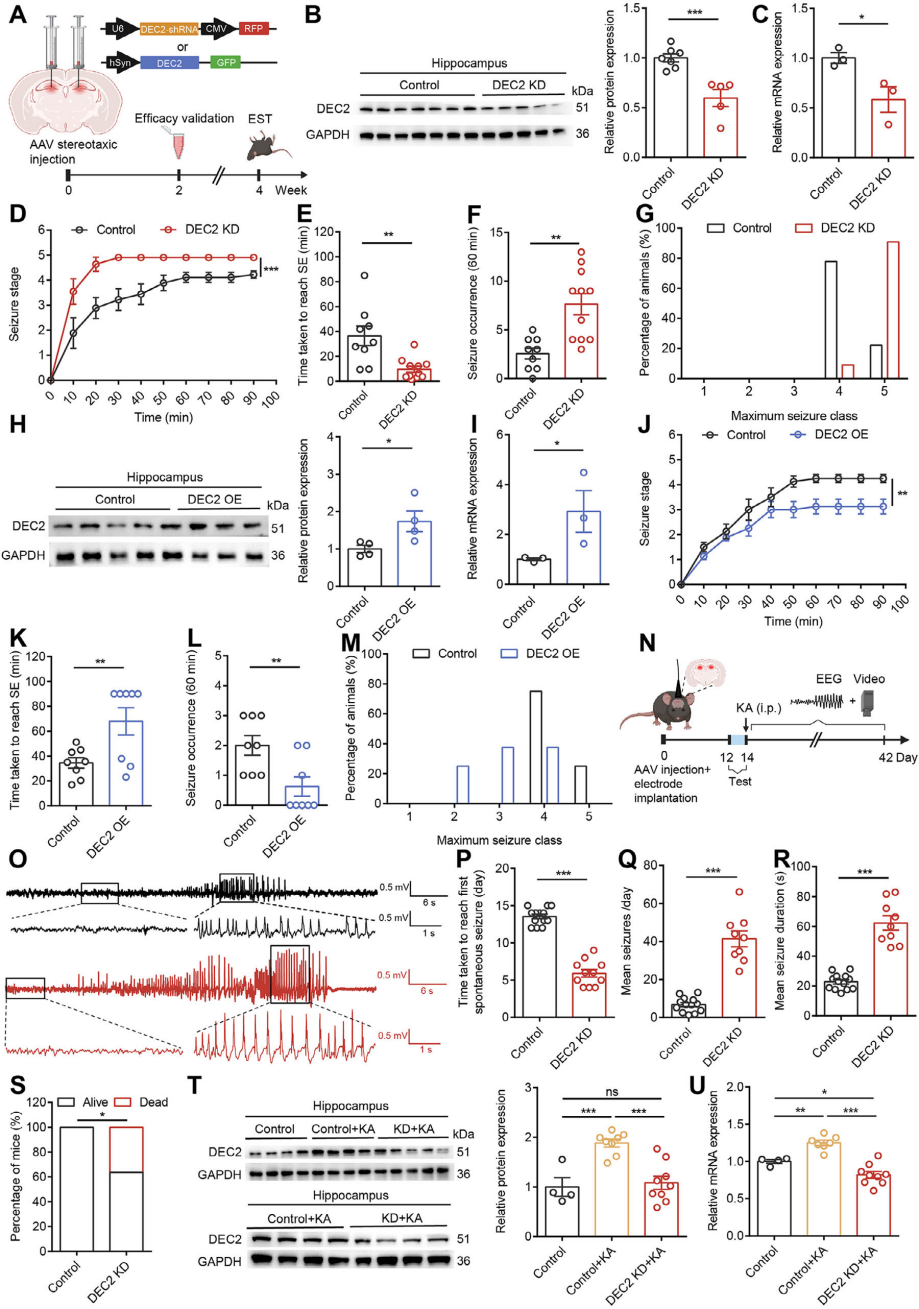
DEC2 suppresses seizure susceptibility and epileptogenesis. A) Schematic diagram of the experimental design for viral stereotactic injection and behavioral tests. B) Western blot and quantification showing DEC2 expression in mouse hippocampus infected with control shRNA and AAV‐DEC2 shRNA‐RFP. n = 7 for control and n = 5 for DEC2 KD, ***p < 0.001, unpaired two‐tailed Student's t test. C) The mRNA levels of *Dec2* in mouse hippocampus infected with control shRNA and AAV‐DEC2 shRNA‐RFP. The levels of mRNA were normalized to that of β‐actin. n = 3 per group, *p < 0.05, unpaired two‐tailed Student's t test. D) Seizure stage illustrated as mean maximum seizure class reached every 10 min following KA administration. n = 9 for control and n = 11 for DEC2 KD, ***p < 0.001, two‐way ANOVA with Bonferroni's multiple‐comparisons test. E–G) Time taken to reach status epilepticus (E), seizure occurrence (F) and incidence of maximum seizure class reached (G) during the experiment. n = 9 for control and n = 11 for DEC2 KD, **p < 0.01, unpaired two‐tailed Student's t‐test. H) Western blot and quantification showing DEC2 expression in mouse hippocampus infected with AAV‐control and AAV‐DEC2‐GFP. n = 4 per group, *p < 0.05, unpaired two‐tailed Student's t test. I) The mRNA levels of *Dec2* in mouse hippocampus infected with AAV‐control and AAV‐DEC2‐GFP. The levels of mRNA were normalized to that of β‐actin. n = 3 per group, *p < 0.05, unpaired two‐tailed Student's t test. J) Seizure stage illustrated as mean maximum seizure class reached every 10 min following KA administration. n = 8, **p < 0.01, two‐way ANOVA with Bonferroni's multiple‐comparisons test. K–M) Time taken to reach status epilepticus (K), seizure occurrence (L) and incidence of maximum seizure class reached (M) during the experiment. n = 8, **p < 0.01, unpaired two‐tailed Student's t‐test. N) Schematic diagram of the experimental design for EEG‐video monitoring in the chronic seizure model. O) Examples of in vivo EEG recordings obtained from control (black) and DEC2 KD (red) mice. P) Time taken to reach first spontaneous seizure after administration of KA from control and DEC2 KD mice. n = 13 for control and n = 11 for DEC2 KD, ***p < 0.001, unpaired two‐tailed Student's t‐test. Q) Quantification of mean seizures per day. n = 13 for control and n = 9 for DEC2 KD, ***p < 0.001, unpaired two‐tailed Student's t‐test. R) Quantification of mean seizure duration. n = 13 for control and n = 9 for DEC2 KD, ***p < 0.001, unpaired two‐tailed Student's t‐test. S) Mortality of DEC2 KD mice (n = 11) and control (n = 13) mice during the chronic phase of epilepsy. *p < 0.05, Chi‐square test. T) Western blot and quantification showing DEC2 expression in the hippocampus of mouse injected with control shRNA and AAV‐DEC2 shRNA‐RFP, analyzed four weeks post‐KA injection. n = 4 for control, n = 8 for control + KA, and n = 9 for DEC2 KD + KA, ***p < 0.001, one‐way ANOVA with Bonferroni's multiple‐comparisons test. U) The mRNA levels of *Dec2* in the hippocampus of mouse injected with control shRNA and AAV‐DEC2 shRNA‐RFP, analyzed four weeks post‐KA injection. The levels of mRNA were normalized to that of β‐actin. n = 4 for control, n = 7 for control + KA, and n = 9 for DEC2 KD + KA, *p < 0.05, **p < 0.01, ***p < 0.001, one‐way ANOVA with Bonferroni's multiple‐comparisons test. Data were represented as mean ± SEM.

To explore the physiological function of DEC2 in mouse brain, we first designed and validated adeno‐associated viruses (AAVs) to manipulate DEC2 expression specifically. For the AAV‐mediated knockdown of DEC2, we developed three distinct short hairpin RNA (shRNA) interference sequences. These sequences were cloned into plasmid expression vectors and transfected into HEK293T cells that overexpressed DEC2 to assess their knockdown efficacy. Our RT‐PCR results indicated that all three shRNA sequences effectively decreased DEC2 expression, with shRNA1 showing the highest efficacy by achieving a 94% decrease in *Dec2* mRNA expression levels (Figure S2A, Supporting Information). Therefore, we chose shRNA1 for packaging into the AAV vector, yielding the AAV‐DEC2 shRNA‐RFP (DEC2 KD). This construct incorporated the U6 promoter for driving DEC2‐shRNA expression and the CMV promoter for expressing the RFP. Subsequently, we injected either a non‐silencing control shRNA or AAV‐DEC2 shRNA‐RFP into the hippocampal DG region of mouse. Robust expression of the fluorescent protein was detected from 14 days post‐injection (DPI), which allowed for effective functional validation between 14–21 DPI, as well as behavioral assessments to be conducted at 28 DPI (**Figure** **2**A). We validated the efficacy of AAV‐DEC2 shRNA‐RFP in knocking down DEC2 expression through western blot and RT‐PCR analysis (Figure 2B,C). This approach enabled us to effectively manipulate DEC2 expression in specific brain regions, providing a valuable tool for subsequent functional studies.

To investigate the pathophysiological consequences of aberrant DEC2 expression in vivo, we examined the effects of DEC2 knockdown on seizure susceptibility in an animal model of TLE.

We began by performing stereotaxic injections of either control shRNA or AAV‐DEC2 shRNA‐RFP virus into the DG of the mouse hippocampus. Subsequently, we induced seizure activity by a single intraperitoneal injection of KA at 20 mg kg^−1^. This dose was selected based on established literature demonstrating its effectiveness in reliably inducing status epilepticus without causing excessive mortality in mice.^[^ ^18^ ^]^ We assessed the severity of seizures using the modified Racine Scale^[^ ^19^ ^]^ and observed that DEC2 KD mice exhibited an accelerated progression of seizures, indicated by the higher maximum seizure class they reached in each 10‐min interval as compared to the control mice within the 90‐min observation period (Figure 2D). The severity of seizures in DEC2 KD mice was evident, leading to a 20% mortality rate within the observation period, in contrast to the absence of mortality in the control group. Additionally, DEC2 KD mice exhibited a reduced latency to the onset of status epilepticus (Figure 2E). The average seizure occurrence and the maximum seizure severity were markedly elevated in the DEC2 KD mice, with a higher proportion progressing to seizure stage five, characterized by generalized tonic‐clonic convulsions (Figure 2F,G). In addition, to investigate whether the knockdown of DEC2 had any effect on other neuropsychiatric behaviors, we performed a battery of behavioral tests, including the open field test (OFT) and the elevated plus maze test (EPM) to evaluate locomotor activity and anxiety‐related behavior, as well as the sucrose preference test (SPT) and the tail suspension test (TST) to analyze depression‐related behavior (Figure S2B, Supporting Information). Behaviorally, DEC2 KD mice showed no differences in the distance traveled or the time in the center in the OFT, and no disparities in the number of entries or the time spent in the open arms in the EPM when compared to the control mice (Figure S2C,D, Supporting Information). Additionally, DEC2 KD mice displayed a similar sucrose preference ratio in the SPT and consistent levels of total immobility time in the TST compared to the control mice (Figure S2E,F, Supporting Information). In summary, these findings suggest that knockdown of DEC2 significantly increases susceptibility to epileptic seizures and exacerbates their severity, while exerting no effect on locomotor activity, anxiety‐related behavior, or depression‐related behavior.

Having examined the consequences of DEC2 knockdown, we next sought to investigate the effects of DEC2 overexpression on seizure susceptibility. To this end, we employed an AAV‐mediated overexpression strategy, cloning the full‐length *Dec2* gene into an AAV vector to generate AAV‐DEC2‐GFP (DEC2 OE). This construct was designed with the human synapsin (hSyn) promoter to drive the expression of the *Dec2* coding sequence, along with the GFP reporter gene, thereby ensuring neuron‐specific expression. We validated the efficacy of AAV‐DEC2‐GFP through western blot and RT‐PCR analysis (Figure 2H,I). Subsequently, we administered the AAV‐DEC2‐GFP virus into the hippocampal DG region of mouse to induce overexpression of DEC2. We observed that mice with elevated levels of DEC2 in their hippocampus displayed a significant reduction in their susceptibility to induced seizures. These mice exhibited a delayed progression of seizures, a prolonged latency to reach status epilepticus, and a decrease in seizure severity when compared to the control mice (Figure 2J–M). Moreover, the behavioral assessments revealed that DEC2 OE mice showed no alterations in locomotor activity, anxiety‐ or depression‐related behaviors (Figure S2G–J, Supporting Information). Collectively, these findings indicate that DEC2 overexpression mitigates seizure susceptibility and severity, supporting its protective role in the brain.

To examine whether changes in DEC2 expression influenced the progression of chronic seizures, we employed a well‐established KA‐induced chronic seizure model in C57BL/6 mice. Prior to seizure induction, we selectively knocked down DEC2 expression levels in the hippocampal DG through stereotaxic delivery of AAV‐DEC2 shRNA‐RFP. Following KA administration (25 mg kg^−1^, i.p.), we terminated status epilepticus with sodium pentobarbital (SP) (37 mg kg^−1^, i.p.) and performed long‐term electroencephalography (EEG)‐video monitoring for 28 days to track seizure development (Figure 2N). Spontaneous seizures typically occurred 5–30 days post‐SE, with an average of 14 days,^[^ ^20^ ^]^ closely matching the 13.54‐day latency observed in our control mice. Compared to control mice, DEC2 KD mice displayed a significant reduction in seizure latency (13.54 ± 0.31 versus 5.91 ± 0.51 days, p < 0.001***), indicating an accelerated onset of spontaneous seizures (Figure 2O,P). To assess changes in spontaneous seizure dynamics during the chronic phase of epilepsy, we quantified chronic spontaneous convulsive seizures during the 2–3 weeks post KA injection. DEC2 KD mice showed a higher daily seizure frequency (6.79 ± 1.07 v 41.41 ± 4.20 seizures/day, p < 0.001***) and prolonged seizure duration per event (22.84 ± 1.45 vs 62.28 ± 4.76 s, p < 0.001***) (Figure 2Q,R), suggesting increased epileptogenic activity. In addition, DEC2 KD mice showed higher mortality in the latent period of epilepsy following SE induction (0 vs 36.36%, p < 0.05*) (Figure 2S), reinforcing the neuroprotective role of DEC2 in epilepsy progression. Post‐hoc hippocampal analysis confirmed sustained DEC2 upregulation in the chronic phase of KA‐induced epilepsy, as evidenced by significantly elevated protein and mRNA levels 28 days post KA. Importantly, AAV‐DEC2 shRNA‐RFP administration effectively reversed this elevation in epileptic mice, with significant reductions in both DEC2 protein and mRNA expression levels in KA‐injected DEC2 KD mice, confirming effective knockdown (Figure 2T,U). These findings indicate that DEC2 plays a crucial role in mitigating epilepsy progression by delaying seizure onset and reducing seizure severity. These results further support the notion that DEC2 suppresses epileptogenesis.

### 2.3 DEC2 Inhibits Intrinsic Excitability and Excitatory Synaptic Activity of Hippocampal Neurons

**Figure 3:**
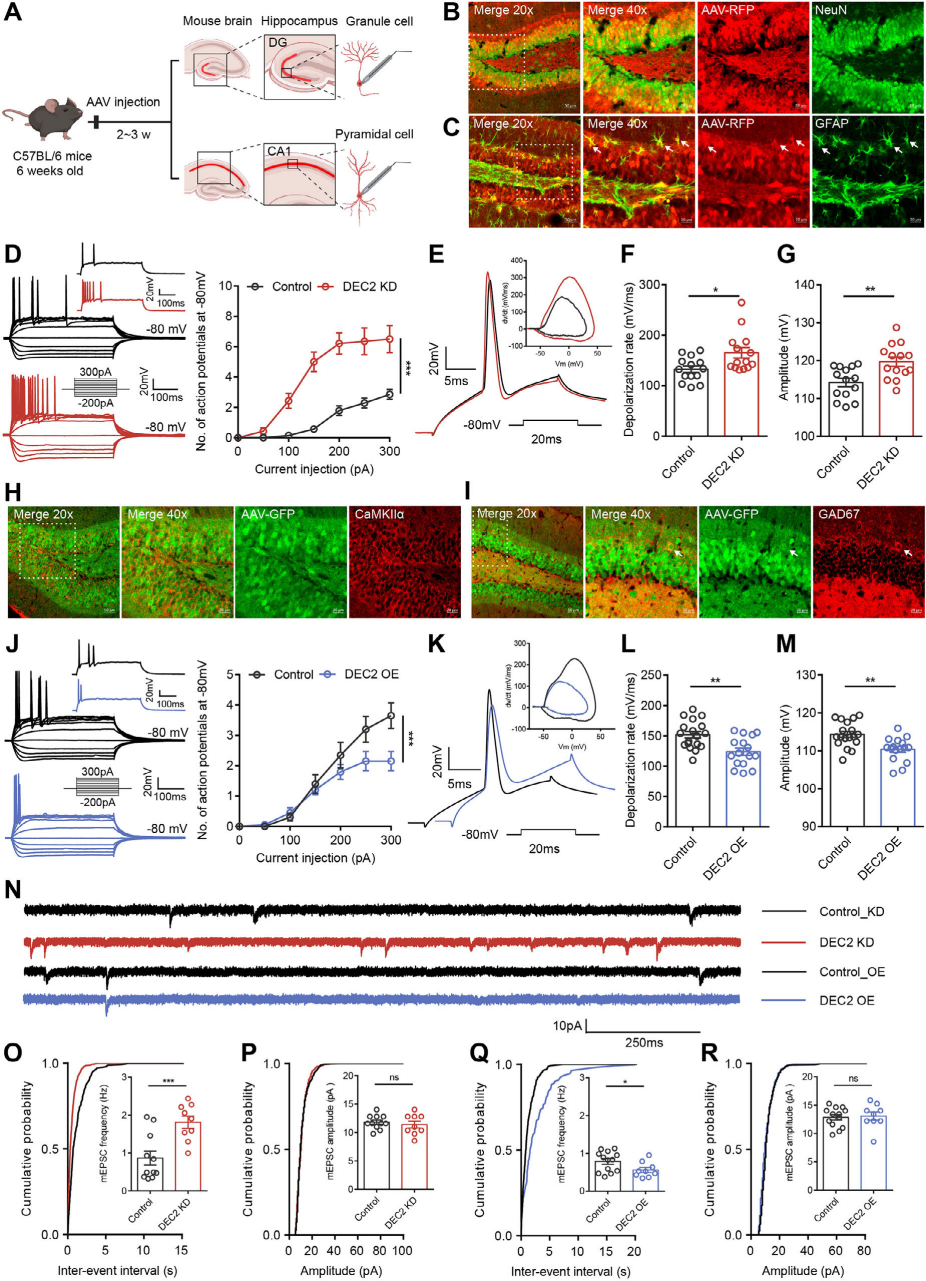
DEC2 inhibits intrinsic excitability and excitatory synaptic activity of hippocampal neurons. A) Schematic diagram of the experimental design for electrophysiological recordings. B) Representative images showing AAV‐DEC2 shRNA‐RFP (red) co‐localization with NeuN (green) at 20 × (left panel, scale bar = 50 µm) and 40 × magnification (right three panels: merged view of boxed area, AAV‐DEC2 shRNA‐RFP single channel, and NeuN single channel; scale bar = 20 µm). C) Representative images showing AAV‐DEC2 shRNA‐RFP (red) co‐localization with GFAP (green) at 20 × (left panel, scale bar = 50 µm) and 40 × magnification (right three panels: merged view of boxed area, AAV‐DEC2 shRNA‐RFP single channel, and GFAP single channel; scale bar = 20 µm). White arrows indicate GFAP‐positive cells. D) Representative current‐clamp recordings and the mean number of action potentials generated in response to depolarizing current pulses obtained from DG neurons of control (black) and DEC2 KD (red) mice. n = 14 per group, ***p < 0.001, two‐way ANOVA with Bonferroni's multiple‐comparisons test. E–G) Typical spikes and associated phase‐plane plots (E), depolarization rate (F), and amplitude (G) of action potentials obtained from DG neurons of control and DEC2 KD mice. n = 14 per group, *p < 0.05, **p < 0.01, unpaired two‐tailed Student's t‐test. H) Representative images showing AAV‐DEC2‐GFP (green) co‐localization with CaMKIIα (red) at 20 × (left panel, scale bar = 50 µm) and 40 × magnification (right three panels: merged view of boxed area, AAV‐DEC2‐GFP single channel, and CaMKIIα single channel; scale bar = 20 µm). I) Representative images showing AAV‐DEC2‐GFP (green) co‐localization with GAD67 (red) at 20 × (left panel, scale bar = 50 µm) and 40 × magnification (right three panels: merged view of boxed area, AAV‐DEC2‐GFP single channel, and GAD67 single channel; scale bar = 20 µm). White arrows indicate GAD67‐positive cells. J) Representative current‐clamp recordings and the mean number of action potentials generated in response to depolarizing current pulses obtained from DG neurons of control (black) and DEC2 OE (blue) mice. n = 20 per group, ***p < 0.001, two‐way ANOVA with Bonferroni's multiple‐comparisons test. K–M) Typical spikes and associated phase‐plane plots (K), depolarization rate (L), and amplitude (M) of action potentials obtained from DG neurons of control and DEC2 OE mice. n = 18 for control and n = 16 for DEC2 OE, **p < 0.01, unpaired two‐tailed Student's t‐test. N) Representative mEPSC recording traces. O) Cumulative distributions of the mEPSC interevent intervals and quantifications of mEPSC frequency from DG neurons of control (n = 11) and DEC2 KD (n = 9) mice. ***p < 0.001, unpaired two‐tailed Student's t‐test. P) Cumulative distributions of the mEPSC amplitudes and quantifications of mEPSC amplitudes from DG neurons of control (n = 11) and DEC2 KD (n = 9) mice. Unpaired two‐tailed Student's t‐test. Q) Cumulative distributions of the mEPSC interevent intervals and quantifications of mEPSC frequency from DG neurons of control (n = 13) and DEC2 OE (n = 9) mice. *p < 0.05, unpaired two‐tailed Student's t‐test. R) Cumulative distributions of the mEPSC amplitudes and quantifications of mEPSC amplitudes from DG neurons of control (n = 13) and DEC2 OE (n = 9) mice. Unpaired two‐tailed Student's t‐test. Data were represented as mean ± SEM.

Epilepsy is characterized by impaired neuronal plasticity. To examine the influence of DEC2 on neuronal plasticity, we first validated the specificity and efficiency of our viral transduction approach. Stereotaxic injection of AAV‐DEC2 shRNA‐RFP (U6 promoter) into the DG showed robust co‐localization with both neuronal (NeuN^+^) and astrocytic (GFAP^+^) markers at 14 DPI (**Figure** **3**A–C), confirming broad transduction across neural cell types as expected from the ubiquitous U6 promoter. To assess the impact of DEC2 knockdown on intrinsic plasticity, we measured the intrinsic excitability of hippocampal DG neurons through whole‐cell current‐clamp electrophysiological recordings from control and DEC2 KD mice at 14–21 DPI. We identified hippocampal DG granule neurons based on their localization, morphological properties, and input resistances (RN) of 193.30 ± 6.69 MΩ (n = 56), in line with previous reports.^[^ ^21^ ^]^ Neurons were held at a membrane potential of −80 mV, and incremental steps of current injection were applied to record the number of APs elicited. We observed a significantly increased number of APs in DEC2 KD mice (Figure 3D), indicating that DEC2 knockdown enhances neuronal excitability. Similar results were obtained when neurons were held at their normal resting membrane potential (RMP) (Figure S3A, Supporting Information). Furthermore, we applied a minimal positive current injection to hippocampal DG granule neurons to induce a single AP. To visualize the fast dynamics of action potentials, we plotted AP velocity against voltage in a phase‐plane plot, of which the rising phase of the AP indicated the recruitment of the AIS and somatic sodium channels.^[^ ^8b^ ^]^ In DG granule neurons of DEC2 KD mice, the rising phase dV/dt was faster (Figure 3E), suggesting a more robust activation of voltage‐gated sodium currents (I_Na_) compared to the control mice. Additionally, DEC2 knockdown resulted in a significant increase in the depolarization rate (mV/ms) and the amplitude of APs, without altering the AP threshold (Figure 3F,G; and Table S2, Supporting Information). To more fully characterize the molecular mechanism responsible for the altered AP output in DEC2 KD neurons, we measured the afterhyperpolarization (AHP), a key regulator of neuronal spike frequency. The AHP is primarily mediated by calcium‐activated potassium channels and sodium‐activated potassium channels, which influence the membrane hyperpolarization, thereby affecting the likelihood of neurons reaching the threshold for subsequent APs.^[^ ^22^ ^]^ To assess this, we held DG neurons at a membrane potential of −80 mV and stimulated them with a 100 Hz current pulse train, resulting in a summed AHP. Our findings revealed that the summed AHP was unchanged between the control and DEC2 KD neurons (Figure S3B, Supporting Information). These results indicate that DEC2 regulates neuronal output spiking through its control of the depolarization and amplitude of APs.

To further examine the function of DEC2 on intrinsic excitability in vivo, we conducted overexpression experiments by specifically delivering either control virus or AAV‐DEC2‐GFP to the mouse hippocampal DG region. Immunofluorescence analysis confirmed efficient AAV‐DEC2‐GFP transduction in the DG, with the Syn promoter‐driven construct showing selective neuronal tropism. Notably, the GFP co‐localized with markers of both excitatory (CaMKIIα^+^) and inhibitory (GAD67^+^) neurons (Figure 3H,I), confirming targeted expression in the intended cell populations without disrupting endogenous DEC2 distribution. In contrast to the enhanced excitability observed in DEC2 KD neurons, DEC2 OE neurons exhibited decreased neuronal excitability, as demonstrated by fewer APs generated during current injection (Figure 3J). Electrophysiological analysis revealed that DEC2 OE neurons displayed a slower rising phase dV/dt compared to controls, concurrent with a decrease in both the depolarization rate (mV/ms) and AP amplitude, with the AP threshold remaining unchanged (Figure 3K–M; Figure S3C,D and Table S3, Supporting Information). To exclude potential regional specificity within the brain, we manipulated DEC2 expression levels in the CA1 region of the mouse hippocampus. We identified hippocampal CA1 pyramidal neurons based on their localization, morphological properties, and RN of 157.80 ± 7.42 MΩ (n = 10), consistent with previous reports.^[^ ^23^ ^]^ Electrophysiological recordings in the CA1 region confirmed our findings, showing that DEC2 knockdown enhanced intrinsic neuronal excitability, whereas DEC2 overexpression diminished it (Figure S3E–L and Table S4, Supporting Information). Altogether, these data indicate that DEC2 inhibits intrinsic neuronal excitability in vivo.

To investigate alterations in synaptic plasticity in DEC2 KD mice, we analyzed miniature excitatory postsynaptic currents (mEPSCs) in virus‐infected DG granule neurons in hippocampal slices in the presence of tetrodotoxin (TTX, 0.5 µm), bicuculline (10 µm), and CGP 55 845 (1 µm) at 14–21 DPI. The frequency of mEPSCs was increased in DEC2 KD neurons compared to controls, whereas in DEC2 OE neurons, the frequency was decreased, with no alterations in amplitudes observed among all groups (Figure 3N–R). The lack of alteration in mEPSC amplitudes in DG granule neurons suggests that postsynaptic receptor density or sensitivity is not affected, and the changes in synaptic transmission are primarily due to presynaptic mechanisms. We also analyzed miniature inhibitory postsynaptic currents (mIPSCs) in DG granule neurons in the presence of TTX (0.5 µm), APV (50 µm), and CNQX (10 µm), and found no differences in amplitudes and frequency across all groups (Figure S4A–E, Supporting Information), indicating little effect of DEC2 on inhibitory synaptic transmission. These findings suggest that the increased excitability observed in the DEC2 KD neurons is primarily attributable to an increase in excitatory input to the principal neurons. Overall, our findings establish DEC2 as a key regulator of both intrinsic excitability and excitatory synaptic drive in hippocampal circuits.

### 2.4 Genome‐Wide Identification of DEC2 Target Genes in Mouse Hippocampus

**Figure 4:**
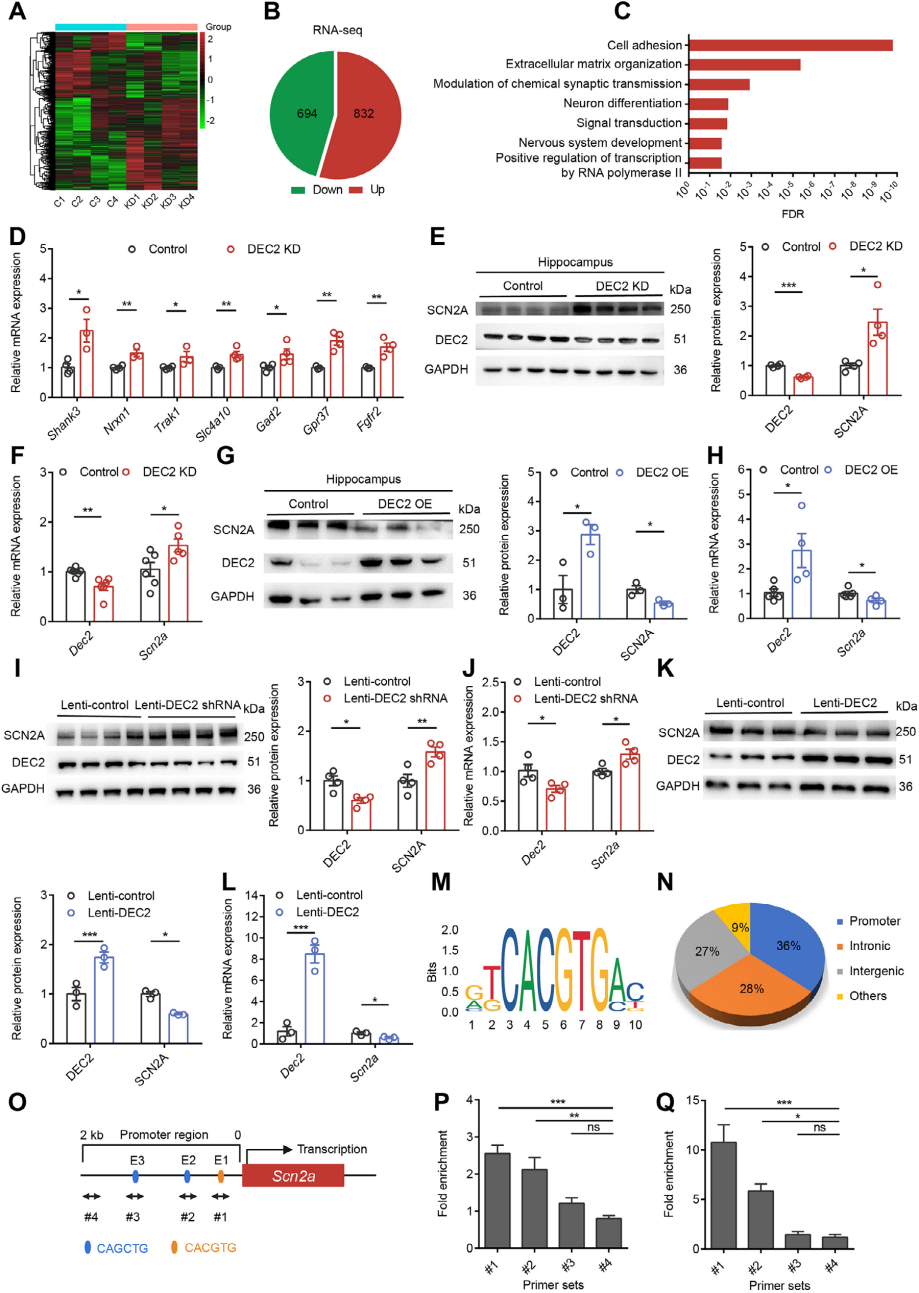
Identification of *Scn2a* as a target gene of DEC2 in hippocampal neurons. A) RNA‐seq analysis of the hippocampal transcriptome from control and DEC2 KD mice. The heatmap displayed differentially expressed genes, with upregulated genes indicated in red and downregulated genes in green. n = 4 per group. B) Downregulated (Down) or upregulated (Up) genes in the hippocampus of DEC2 KD mice compared with controls, as identified by RNA‐seq analysis. C) GO analysis of the upregulated genes in the hippocampus of DEC2 KD mice. D) RT‐PCR measuring the mRNA levels of indicated genes. The levels of mRNA were normalized to that of β‐actin. n = 3 to 4 for each group. *p < 0.05, **p < 0.01, unpaired two‐tailed Student's t‐test. E) Western blot and quantification showing that knockdown of DEC2 increased SCN2A expression in mouse hippocampus. n = 4 per group, *p < 0.05, ***p < 0.001, unpaired two‐tailed Student's t‐test. F) Knockdown of DEC2 increased *Scn2a* mRNA levels in mouse hippocampus. The levels of mRNA were normalized to that of β‐actin. n = 6 for control and n = 5 for DEC2 KD, *p < 0.05, **p < 0.01, unpaired two‐tailed Student's t‐test. G) Western blot and quantification showing that overexpression of DEC2 decreased SCN2A expression in mouse hippocampus. n = 3 per group, *p < 0.05, unpaired two‐tailed Student's t‐test. H) Overexpression of DEC2 decreased *Scn2a* mRNA levels in mouse hippocampus. The levels of mRNA were normalized to that of β‐actin. n = 5 for control and n = 4 for DEC2 OE, *p < 0.05, unpaired two‐tailed Student's t‐test. I) Western blot and quantification showing that knockdown of DEC2 increased SCN2A expression in mouse primary cortical neurons. n = 4 per group, *p < 0.05, **p < 0.01, unpaired two‐tailed Student's t‐test. J) Knockdown of DEC2 increased *Scn2a* mRNA levels in mouse primary cortical neurons. The levels of mRNA were normalized to that of β‐actin. n = 4 per group, *p < 0.05, unpaired two‐tailed Student's t‐test. K) Western blot and quantification showing that overexpression of DEC2 decreased SCN2A expression in mouse primary cortical neurons. n = 3 per group, *p < 0.05, ***p < 0.001, unpaired two‐tailed Student's t‐test. L) Overexpression of DEC2 decreased *Scn2a* mRNA levels in mouse primary cortical neurons. The levels of mRNA were normalized to that of β‐actin. n = 3 per group, *p < 0.05, ***p < 0.001, unpaired two‐tailed Student's t‐test. M) Vertebrate DEC2 consensus binding motif. Motif screening was performed using JASPAR.^[^ ^25^ ^]^ N) Genomic distribution of DEC2 binding regions determined by ChIP‐seq analysis.^[^ ^26^ ^]^ O) Schematized model of the mouse *Scn2a* gene promoter, with the translation start site designated as the zero point. P) DEC2 bound to the promoter region of the *Scn2a* gene in mouse hippocampus. qChIP assays were performed with primer pairs specific to indicated regions. n = 4 for each group, **p < 0.01, ***p < 0.001, one‐way ANOVA with Bonferroni's multiple‐comparisons test. Q) DEC2 bound to the promoter region of the *Scn2a* gene in mouse primary cortical neurons. n = 3 for each group, *p < 0.05, ***p < 0.001, one‐way ANOVA with Bonferroni's multiple‐comparisons test. Data were represented as mean ± SEM.

To elucidate the molecular mechanisms by which DEC2 regulates neuronal plasticity and epileptogenesis, we performed genome‐wide RNA sequencing (RNA‐seq) on hippocampal tissue infected with control shRNA or AAV‐DEC2 shRNA‐RFP virus (**Figure** **4**A). Using a *p*‐value threshold (*p* < 0.05), we identified 832 upregulated and 694 downregulated differentially expressed genes (DEGs) (Figure 4B). Given that DEC2 primarily functions as a transcriptional repressor, we directed our attention to the upregulated DEGs in DEC2 KD mice, as these are more likely to be direct targets of DEC2 repression. Notably, multiple enriched Gene Ontology (GO) pathways are related to key mechanisms underlying neuronal function and plasticity, including cell adhesion, extracellular matrix organization, modulation of chemical synaptic transmission, neuron differentiation, signal transduction, and nervous system development pathway (Figure 4C; Tables S5 and S10, Supporting Information), suggesting DEC2's role in maintaining synaptic architecture and function. To validate the gene expression changes identified by RNA‐seq and to rule out potential batch effects, we conducted RT‐PCR on hippocampal tissue from DEC2 KD mice, including *Scn2a*, *Shank3*, and *Slc4a10*, and confirmed the altered expression of these genes in the expected direction (Figure 4D). Therefore, these results supported the reliability and reproducibility of the RNA‐seq data. Notably, we observed that *Scn2a*, an ion channel gene encoding the voltage‐gated sodium channel Na_V_1.2, was significantly upregulated in DEC2 KD mice compared to the controls. SCN2A has previously been implicated in regulating intrinsic neuronal excitability and synaptic function,^[^ ^8^, ^10^, ^24^ ^]^ making it a strong candidate gene for our observed phenotype.

To determine whether DEC2 regulates SCN2A expression, we performed knockdown and overexpression experiments in both mouse hippocampal tissue and primary neuronal cultures. In the hippocampus, knockdown of DEC2 significantly increased SCN2A expression, while overexpression of DEC2 led to a decrease in SCN2A expression at both the protein and mRNA levels (Figure 4E–H). Importantly, this regulation was specific to SCN2A, as RT‐PCR analysis showed no significant changes in other ion channel genes associated with the depolarization and repolarization of APs (Figure S4F, Supporting Information). To validate these findings in a defined cellular system, we established primary cortical neuron cultures from postnatal day 0 C57BL/6 mice. Following lentiviral transduction at postnatal day 3 (P3) with either DEC2‐targeting short hairpin RNA (lenti‐DEC2 shRNA) or full‐length *Dec2* gene (lenti‐DEC2), we performed western blot and RT‐PCR analyses 72 h post‐infection. Similarly, neurons infected with lenti‐DEC2 shRNA exhibited increased SCN2A expression, whereas neurons infected with lenti‐DEC2 decreased SCN2A expression (Figure 4I–L), recapitulating our in vivo observations. These results demonstrate that DEC2 specifically modulates SCN2A expression in the central nervous system (CNS).

### 2.5 DEC2 Represses *Scn2a* Transcription

Previous studies have shown that DEC2 primarily functions as a transcriptional repressor.^[^ ^14^, ^26^, ^27^ ^]^ To determine whether DEC2 directly binds to the *Scn2a* genome, we initially analyzed publicly available JASPAR datasets from DEC2 ChIP‐seq experiments performed in human 293T/FT cell line^[^ ^25^ ^]^ and characterized it binding motif (Figure 4M). Genomic distribution analysis revealed that DEC2 occupancy was most prominent at the proximal promoter (36%), intronic (28%), and intergenic regions (27%)^[^ ^26^ ^]^ (Figure 4N). Upon analyzing the 2‐kb promoter region upstream of the mouse *Scn2a* transcription start site, we identified three E‐box elements (CANNTG) that serve as potential DEC2 binding sites, located at positions −99 to −93 base pairs (CACGTG, E‐box 1), −174 to −168 base pairs (CAGCTG, E‐box 2), and −1388 to −1382 base pairs (CAGCTG, E‐box 3) (Figure 4O). To establish whether DEC2 binds to the *Scn2a* promoter, we performed chromatin immunoprecipitation (ChIP) coupled with RT‐PCR in both mouse hippocampal tissue and primary cortical neurons. To examine DEC2 binding in vivo, we stereotaxically injected AAV‐DEC2‐3xFLAG into the mouse hippocampal DG and allowed 4 weeks for stable viral expression. For subsequent ChIP analysis, we designed specific primer sets covering three genomic regions containing predicted E‐box elements: E‐box 1 (−133 to −32 bp, #1), E‐box 2 (−383 to −106 bp, #2), and E‐box 3 (−1491 to −1313 bp, #3). As a negative control, we targeted a distal promoter region (−1925 to −1801 bp, #4) that is devoid of E‐box motifs. Using anti‐FLAG antibody for immunoprecipitation, we observed significant DEC2 enrichment at E‐box 1 and E‐box 2, but not at E‐box 3 or control regions in hippocampal tissue (Figure 4P). In parallel, we confirmed these findings in an in vitro system using primary cortical neurons transduced with a lentiviral vector encoding the DEC2‐3 × FLAG fusion protein, which recapitulated the identical binding specificity (Figure 4Q). These results demonstrate that DEC2 specifically binds to selective E‐box elements within the *Scn2a* promoter to exert its regulatory role.

**Figure 5:**
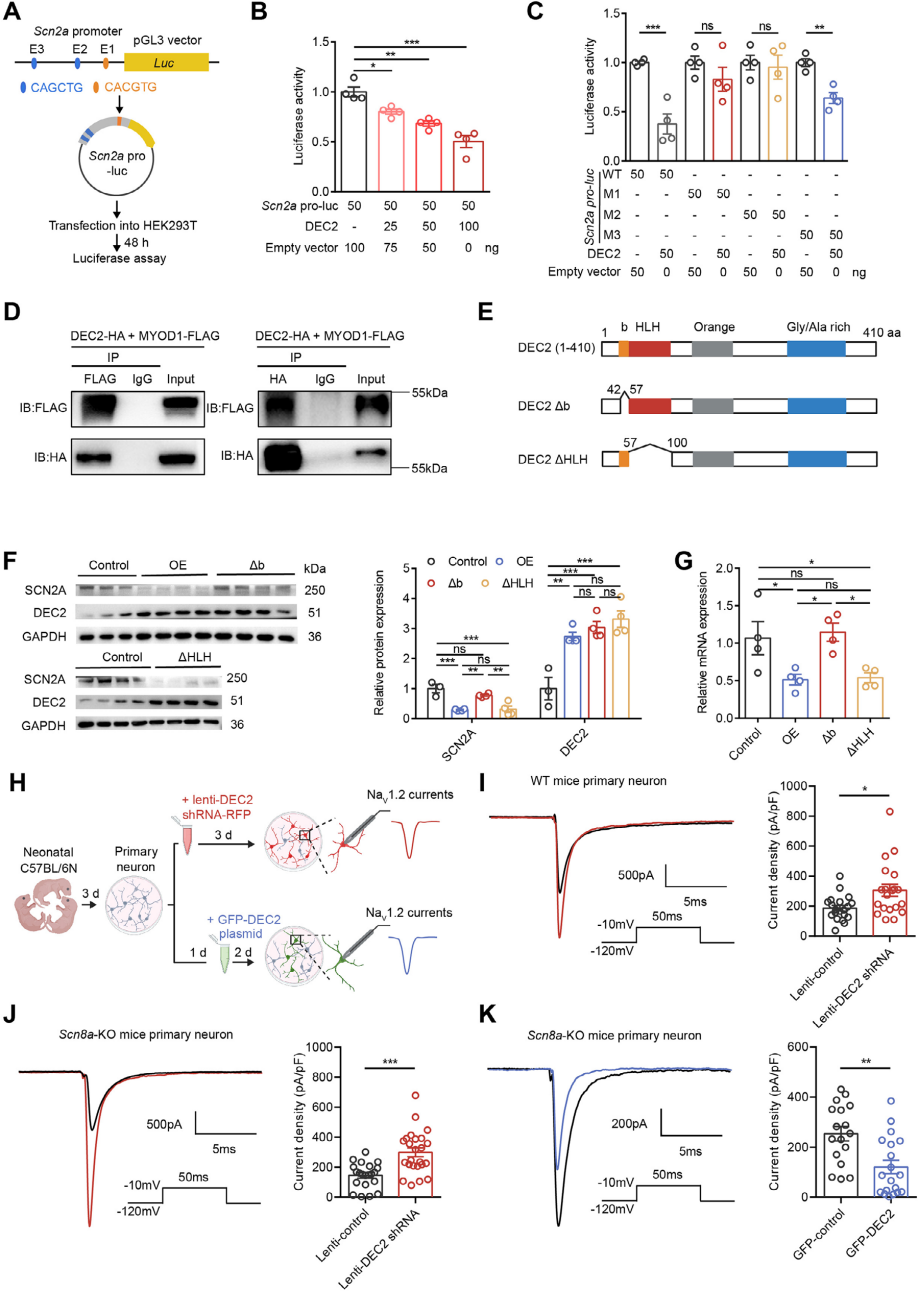
DEC2 represses the transcription of *Scn2a*. A) Schematic diagram of the experimental design for luciferase assay. B) Luciferase assay in HEK293T cells. DEC2 expression inhibits *Scn2a* pro‐luc activity. n = 4 per group, *p < 0.05, **p < 0.01, ***p < 0.001, one‐way ANOVA with Bonferroni's multiple‐comparisons test. C) Luciferase assay using *Scn2a*‐pro luc E‐box mutant constructs. DEC2 repressor activity was not observed for E‐box mutant M1 and M2. n = 4, **p < 0.01, ***p < 0.001, unpaired two‐tailed Student's t‐test. D) Immunoblot analysis of DEC2‐HA and MYOD1‐FLAG in IP‐FLAG (Left) and IP‐HA (Right) samples prepared from HEK293T cell lysate. E) Schematic representation of full‐length DEC2 (1‐410) and its deletion mutants. b, basic region; HLH, helix‐loop‐helix domain; O, orange domain. Numbers indicate the positions of amino acids in the DEC2 protein. F) Western blot and quantification of DEC2 and SCN2A expression in mouse primary cortical neurons following lentiviral infection with control, intact *Dec2* (OE), and *Dec2* deletion mutants. n = 3 for control and n = 4 for other groups, **p < 0.01, ***p < 0.001, one‐way ANOVA with Bonferroni's multiple‐comparisons test. G) The mRNA levels of *Scn2a* in mouse primary cortical neurons following lentiviral infection with control, intact *Dec2* (OE), and *Dec2* deletion mutants. The levels of mRNA were normalized to that of β‐actin. n = 4 per group, *p < 0.05, one‐way ANOVA with Bonferroni's multiple‐comparisons test. H) Schematic diagram of the experimental design for Na_V_1.2 current recording. I) Representative traces of Na_V_1.2 current recorded from control and lenti‐DEC2 shRNA infected wild‐type mice primary cortical neurons (left) and quantification of current density (right). n = 20 per group, *p < 0.05, unpaired two‐tailed Student's t‐test. J) Representative traces of Na_V_1.2 current recorded from control (n = 21) and lenti‐DEC2 shRNA (n = 24) infected *Scn8a*‐KO mice primary cortical neurons (left) and quantification of current density (right). ***p < 0.001, unpaired two‐tailed Student's t‐test. K) Representative traces of Na_V_1.2 current recorded from control (n = 17) and GFP‐DEC2 (n = 19) infected *Scn8a*‐KO mice primary cortical neurons (left) and quantification of current density (right). **p < 0.01, unpaired two‐tailed Student's t‐test. Data were represented as mean ± SEM.

To investigate the transcriptional regulation of SCN2A by DEC2, we conducted luciferase reporter assays using a 1.5‐kb promoter fragment of the *Scn2a* promoter, which includes all three E‐box elements. This promoter fragment was cloned into the pGL3 vector to generate the *Scn2a* pro‐luc reporter construct, which was then transfected into HEK293T cells (**Figure** **5**A). Our results showed that increasing amounts of DEC2 resulted in a progressive reduction in *Scn2a* pro‐luc activity, suggesting that DEC2 functions as a transcriptional repressor of SCN2A expression (Figure 5B). To evaluate the relative contribution of individual E‐box motifs to the regulation of SCN2A by DEC2, we introduced mutated E‐box sequences in the *Scn2a* promoter. Specifically, we mutated E‐box 1 (CACGTG to ACCGGT, designated as M1), E‐box 2 (CAGCTG to ACGCGT, designated as M2), and E‐box 3 (CAGCTG to ACGCGT, designated as M3). Luciferase assays using these mutants revealed that the repression by DEC2 was most pronounced when the E‐boxes were intact or when M3 was present. However, mutations in E‐box1 or E‐box 2 (M1 and M2) significantly attenuated the DEC2 repressor activity of *Scn2a* (Figure 5C), underscoring the significance of E‐box 1 and E‐box 2 elements in DEC2's function. DEC2 employs multiple molecular mechanisms of transcriptional regulation.^[^ ^13^, ^28^ ^]^ As a basic helix‐loop‐helix transcription factor, DEC2 proteins can form homodimers and directly bind to class B E‐boxes (CACGTG) in target gene promoters. Furthermore, DEC2 can also form complexes with other bHLH activator proteins, such as MYOD1, and indirectly bind to another type of E‐box motif (CAGCTG), which is also present in the *Scn2a* promoter region, thereby inhibiting the transactivation of MYOD1. To investigate the potential role of DEC2‐MYOD1 interaction in regulating SCN2A expression, we first examined whether DEC2 and MYOD1 physically interact. To investigate the interaction between DEC2 and MYOD1, we performed co‐immunoprecipitation (co‐IP) experiments in HEK293T cells co‐transfected with FLAG‐tagged MYOD1 and HA‐tagged DEC2 expression constructs. Cell lysates were immunoprecipitated with either anti‐FLAG or anti‐HA antibodies, followed by western blot analysis with the reciprocal antibody. We found that the immunoprecipitation of MYOD1‐FLAG with anti‐FLAG antibody simultaneously captured HA‐tagged DEC2 in HEK‐293T cells, and vice versa (Figure 5D), confirming the robust physical interaction between DEC2 and MYOD1. The above results suggest that DEC2 may modulate *Scn2a* transcription not only through direct DNA binding but also via protein‐protein interaction with MYOD1. To further investigate which mechanism is more critical for DEC2‐mediated repression, we examined the functional domains in DEC2 required for transcriptional repression from CANNTG elements. The basic region has been shown to be necessary for specific DNA binding, whereas the HLH domain mediates dimerization, including heterodimerization with MYOD1.^[^ ^28^ ^]^ Thus, we targeted either the basic domain or the HLH domain and generated deletions of the DEC2 protein, named DEC2 Δb and DEC2 ΔHLH, respectively (Figure 5E). These constructs were cloned into lentiviral vectors and transduced into mouse primary cortical neurons. Compared to the intact DEC2 proteins (OE), which robustly repressed SCN2A expression, deletion of the HLH domain of DEC2 (DEC2 ΔHLH) had little effect on the repression activity. However, deletion of the basic domain (DEC2 Δb) completely abolished the repression activity (Figure 5F,G). The inability of DEC2 Δb mutant to repress the promoter activity may be due to the inactivity of DNA binding, indicating the direct DNA binding mechanism is necessary for the repression activity of DEC2 on SCN2A expression. In contrast, the indirect interaction with MYOD1 may be less significant in primary cells, potentially due to compensatory mechanisms involving other pathways, such as the low expression level of MYOD1 in the mouse brain or the presence of alternative factors. Together, these results suggest that the direct DNA binding mechanism plays a predominant role in DEC2‐mediated repression of *Scn2a* gene.

The epigenetic removal of acetyl groups from histone tails by histone deacetylases (HDACs) causes a compact chromatin structure that represses gene transcription. Many transcriptional repressors exert their action through recruitment of HDAC activity.^[^ ^29^ ^]^ To explore the involvement of HDACs in DEC2‐mediated repression, we treated HEK293T cells with the HDAC inhibitor Trichostatin A (TSA). The results showed that 100 nm TSA completely abrogated the transcriptional repression mediated by DEC2 (Figure S5A, Supporting Information). This observation indicates that DEC2 represses *Scn2a* transcription in an HDAC‐dependent manner. Collectively, our results demonstrate that DEC2 represses *Scn2a* transcription primarily through direct binding to E‐box elements in the *Scn2a* promoter. Although DEC2 interacts with the transcriptional activator MYOD1, this interaction appears to have minimal impact on *Scn2a* regulation under physiological conditions. Additionally, the HDAC‐dependent nature of this repression suggests an epigenetic component to DEC2's regulatory function. These findings provide insights into the molecular basis of *Scn2a* regulation, which may have broader implications for understanding the control of neuronal excitability and the pathogenesis of epilepsy.

### 2.6 DEC2 Inhibits Functional Na_V_1.2‐Mediated Sodium Currents

To elucidate the functional consequences of DEC2‐mediated transcriptional repression on neuronal excitability, we next investigated how DEC2 regulates Na_V_1.2‐mediated sodium currents in primary cortical neurons. It has been reported that Na_V_1.2 and Na_V_1.6 are the main sodium channel subtypes in the glutamatergic neurons within the brain, with Na_V_1.2 being the only sodium channel isoform at the AIS during the first postnatal week in mice.^[^ ^8^, ^9^ ^]^ Therefore, we employed a developmental time window strategy to isolate Na_V_1.2‐specific currents. We isolated primary cortical neurons from P0 mice, infected them with lenti‐DEC2 shRNA at P3, and conducted whole‐cell voltage‐clamp electrophysiological recordings between P6 and P7 (Figure 5H). Quantitative analysis revealed a significant increase in sodium current density upon DEC2 knockdown (Figure 5I), while the voltage‐dependence of activation remained unchanged (Figure S5B, Supporting Information), indicating that DEC2 modulates Na_V_1.2 current amplitude without affecting channel gating properties.

Considering the collaborative role of Na_V_1.6 and Na_V_1.2 in the initiation and propagation of action potentials, we isolated primary cortical neurons from *Scn8a*‐KO mice, which lack the Na_V_1.6 sodium channel subtype (Figure S5C, Supporting Information), to further eliminate the potential interference of Na_V_1.6 currents. Consistent with our initial findings, DEC2 knockdown in *Scn8a*‐KO neurons similarly increased sodium current density without altering activation kinetics (Figure 5J; Figure S5D, Supporting Information), confirming that DEC2 selectively regulates Na_V_1.2 rather than Na_V_1.6 channels. To further validate DEC2's regulatory role, we overexpressed DEC2 in primary cortical neurons from *Scn8a*‐KO mice through transfection with the GFP‐DEC2 plasmid. We transfected *Scn8a*‐KO neurons with GFP‐DEC2 plasmid at P4, and conducted whole‐cell voltage‐clamp electrophysiological recordings between P6 and P7. Notably, we found that overexpression of DEC2 led to a significant reduction in sodium current density when compared to control neurons (Figure 5K). Taken together, these findings provide clear evidence that DEC2 inhibits the functional Na_V_1.2‐mediated sodium currents in neurons.

### 2.7 DEC2 Suppresses Seizure Susceptibility by Negatively Modulating SCN2A Expression

**Figure 6:**
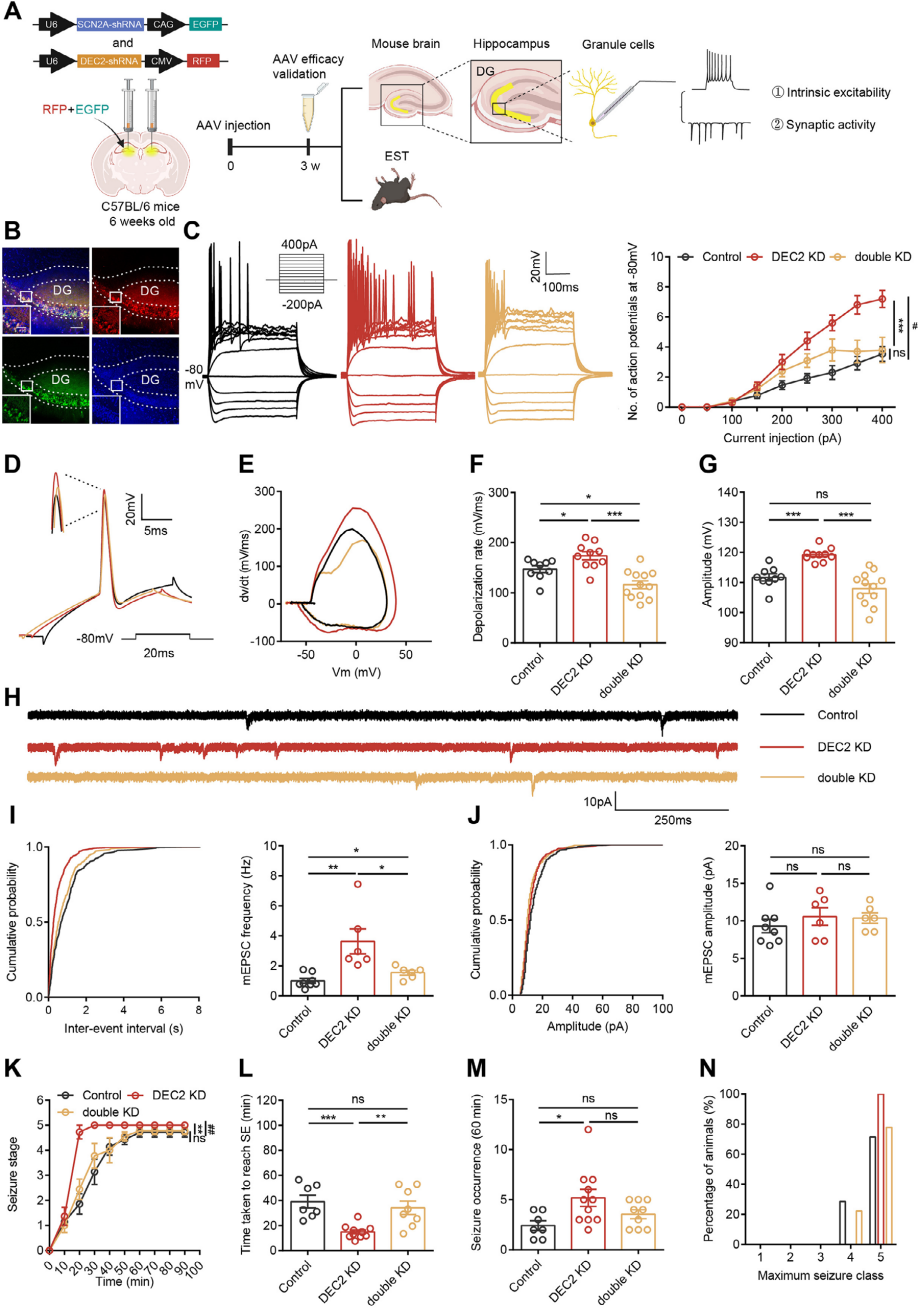
DEC2‐SCN2A pathway regulates epileptogenesis. A) Schematic diagram of the experimental design for viral stereotactic injection, behavioral tests, and electrophysiological recordings. B) Expression of AAV‐SCN2A shRNA‐GFP and AAV‐DEC2 shRNA‐RFP at 21 days after infecting the mouse DG area. Upper left, merge. Bottom left, AAV‐SCN2A shRNA‐GFP infection. Upper right, AAV‐DEC2 shRNA‐RFP infection. Bottom right, DAPI. Scale bar, 50 µm (left), 100 µm (right). C) Representative current‐clamp recordings and the mean number of action potentials generated in response of depolarizing current pulses obtained from DG neurons of control (black), DEC2 KD (red), and double KD (yellow) mice. n = 13 for control and n = 10 for the other group, control versus DEC2 KD*, ***p < 0.001, DEC2 KD versus double KD#, #*p* < 0.05, two‐way ANOVA with Bonferroni's multiple‐comparisons test. D–G) Typical spikes (D), associated phase‐plane plots (E), depolarization rate (F), and amplitude (G) of action potentials obtained from virus‐infected mice DG neurons. n = 9 for control, n = 10 for DEC2 KD, and n = 12 for double KD, *p < 0.05, ***p < 0.001, one‐way ANOVA with Bonferroni's multiple‐comparisons test. H) Representative mEPSC recording traces obtained from hippocampal DG granule neurons of virus‐infected mice. I) Cumulative distributions of the mEPSC interevent intervals and quantifications of mEPSC frequency. n = 8 for control and n = 6 for the other group, *p < 0.05, **p < 0.01, one‐way ANOVA with Bonferroni's multiple‐comparisons test. J) Cumulative distributions of the mEPSC amplitudes and quantifications of mEPSC amplitudes. n = 8 for control and n = 6 for the other group, one‐way ANOVA with Bonferroni's multiple‐comparisons test. K) Graph depicting the seizure progression. n = 7 for control, n = 11 for DEC2 KD and n = 9 for double KD, control versus DEC2 KD*,**p < 0.01, DEC2 KD versus double KD#, ##*p* < 0.01, two‐way ANOVA with Bonferroni's multiple‐comparisons test. L–N) Graph depicting the time taken to reach status epilepticus (L), seizure occurrence (M) and incidence of maximum seizure class reached (N) during the experiment. n = 7 for control, n = 11 for DEC2 KD and n = 9 for double KD, *p < 0.05, **p < 0.01, **p < 0.001, one‐way ANOVA with Bonferroni's multiple‐comparisons test. Data were represented as mean ± SEM.

To investigate whether DEC2‐regulated SCN2A expression underlies the observed neuronal and behavioral phenotypes, we performed a rescue experiment by reducing SCN2A expression in DEC2 knockdown mice. To this end, we constructed an AAV vector expressing SCN2A‐specific shRNA (AAV‐SCN2A shRNA‐GFP) and validated its knockdown efficiency through RT‐PCR analysis (Figure S6A, Supporting Information). Through stereotaxic co‐injection of AAV‐SCN2A shRNA‐GFP and AAV‐DEC2 shRNA‐RFP into the hippocampal DG, we generated double‐knockdown (double KD) mice (**Figure** **6**A,B). Subsequent western blot and RT‐PCR analyses demonstrated that SCN2A knockdown effectively counteracted the elevated SCN2A protein and mRNA levels resulting from DEC2 knockdown (Figure S6B,C, Supporting Information).

We first examined whether SCN2A knockdown could modulate the intrinsic plasticity of hippocampal DG neurons in DEC2 KD mice. Whole‐cell patch‐clamp recordings demonstrated that knockdown of SCN2A in DEC2 KD neurons significantly reduced intrinsic excitability, slowed rising phase dV/dt, and decreased the depolarization rate and amplitude of APs (Figure 6C–G; and Table S6, Supporting Information). Subsequently, we explored whether SCN2A knockdown could also influence the synaptic plasticity of hippocampal DG neurons in DEC2 KD mice. Our findings indicated that SCN2A knockdown in these neurons diminished the elevated frequency of mEPSCs observed in DEC2 KD mice, without altering mEPSC amplitudes among the groups (Figure 6H–J). Additionally, we injected AAV‐SCN2A shRNA‐GFP directly into hippocampal DG neurons and performed whole‐cell current‐clamp recordings. Neurons with SCN2A knockdown exhibited impaired intrinsic excitability and mEPSC frequency (Figure S6D–J and Table S7, Supporting Information), validating the efficacy of this virally mediated gene transfer approach in inhibiting SCN2A function. Together, these findings underscore the critical role of the DEC2‐SCN2A axis in regulating neuronal plasticity.

To determine whether DEC2‐regulated SCN2A expression mediates the hyperexcitability phenotypes in hippocampal neurons, we further examined the effects of SCN2A knockdown on seizure susceptibility in DEC2 KD mice. Remarkably, SCN2A knockdown in the hippocampal DG neurons reversed the behavioral changes in seizure susceptibility and severity that were induced by DEC2 knockdown (Figure 6K–N). Collectively, our data reinforce the pivotal role of the DEC2‐SCN2A axis in the development of epileptogenesis. Altogether, our findings provide compelling evidence that DEC2‐mediated regulation of this critical sodium channel may represent an important mechanism underlying neuronal plasticity and epilepsy pathogenesis.

### 2.8 Cannabidiol Exerts Anticonvulsant Effects via the DEC2‐SCN2A Regulatory Axis

**Figure 7:**
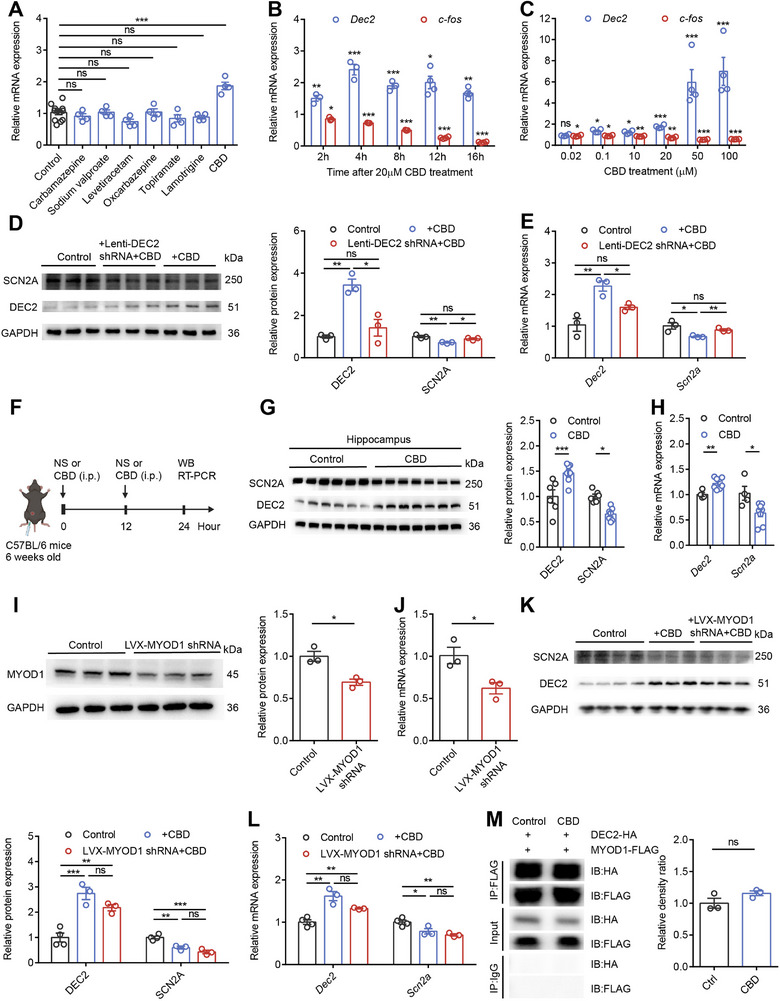
Cannabidiol exerts anticonvulsant effects via the DEC2‐SCN2A axis. A) The mRNA levels of *Dec2* in HT22 cells following 4‐hour treatment with various 20 µm drugs. The levels of mRNA were normalized to that of β‐actin. n = 12 for control and n = 4 for other groups, ***p < 0.001, one‐way ANOVA with Bonferroni's multiple‐comparisons test. B) The mRNA levels of *Dec2* and *c‐fos* in HT22 cells at different time points after 20 µm CBD administration, as determined by RT‐PCR. The levels of mRNA were normalized to that of β‐actin. n = 3 to 4 for each group, *p < 0.05, **p < 0.01, **p < 0.001, two‐way ANOVA with Bonferroni's multiple‐comparisons test. C) The mRNA levels of *Dec2* and *c‐fos* in HT22 cells treated for 4 h with increasing CBD concentrations. The levels of mRNA were normalized to that of β‐actin. n = 4 per group, *p < 0.05, **p < 0.01, **p < 0.001, two‐way ANOVA with Bonferroni's multiple‐comparisons test. D, E) Western blot and RT‐PCR showed that knockdown of DEC2 prevented CBD‐induced DEC2 elevation and SCN2A reduction in HT22 cells. The levels of mRNA were normalized to that of β‐actin. n = 3 per group, *p < 0.05, **p < 0.01, one‐way ANOVA with Bonferroni's multiple‐comparisons test. F) Schematic diagram of the experimental design for in vivo CBD treatment. G,H) Western blot and RT‐PCR showing DEC2 elevation and SCN2A reduction in mouse hippocampus following CBD treatment. The levels of mRNA were normalized to that of β‐actin. n = 6 for control and n = 7 for CBD group in (G), n = 4 for control and n = 8 for CBD group in (H),*p < 0.05, **p < 0.01, **p < 0.001, unpaired two‐tailed Student's t‐test. I,J) Western blot and RT‐PCR showing reduced MYOD1 expression levels in HT22 cells infected with LVX‐MYOD1 shRNA compared to LVX‐control. The levels of mRNA were normalized to that of β‐actin. n = 3 per group, *p < 0.05, unpaired two‐tailed Student's t‐test. K,L) Western blot and RT‐PCR showed that knockdown of MYOD1 had no effect on CBD‐induced DEC2 elevation and SCN2A reduction in HT22 cells. The levels of mRNA were normalized to that of β‐actin. n = 4 for control and n = 3 for other groups, *p < 0.05, **p < 0.01, **p < 0.001, one‐way ANOVA with Bonferroni's multiple‐comparisons test. M) (Left) Immunoblot analysis following immunoprecipitation performed in HEK293T cells. (Right) Quantification of relative density ration between IP and input. n = 3, unpaired two‐tailed Student's t‐test. Data were represented as mean ± SEM.

Our research has demonstrated that upregulating DEC2 in the mouse hippocampus exhibits anticonvulsant protective effects. Given the critical role of DEC2 in modulating neuronal plasticity and seizure susceptibility, we were intrigued by the potential of pharmacological agents to influence DEC2 expression. To identify potential anticonvulsant compounds capable of modulating DEC2 expression, we conducted a pharmacological screening in HT22 mouse hippocampal neuronal cells. The cells were treated for 4 h with 20 µm concentrations of various classical and novel anti‐epileptic drugs, after which *Dec2* mRNA levels were quantitatively analyzed by RT‐PCR. Among all tested compounds, cannabidiol (CBD), a non‐psychoactive cannabinoid, emerged as the most potent inducer of *Dec2* expression (**Figure** **7**A). While CBD has been widely reported for its potential in epilepsy treatment, its precise molecular mechanisms remain incompletely understood. Therefore, we further investigated the impact of CBD on DEC2 expression, aiming to reveal its potential role in the mechanisms of antiepileptic action. To examine the temporal dynamics of CBD's effect on DEC2 expression, we treated HT22 cells with 20 µm CBD and measured *Dec2* mRNA levels at various time points (2, 4, 8, 12, and 16 h). Our analysis showed that CBD treatment led to a rapid and sustained upregulation of *Dec2* expression, with significant increases detectable as early as 2 h post‐treatment and remained stable up to 16 h. This prolonged effect suggests that CBD induces a stable increase in *Dec2* expression, which may contribute to its anticonvulsant properties. Additionally, we used *c‐fos* as an indicator of neuronal activity and observed a corresponding time‐dependent decrease following CBD treatment, indicating a consistent reduction in neuronal excitability that parallels the *Dec2* upregulation (Figure 7B). To further characterize the concentration dependence of CBD's effects on DEC2 expression, we treated HT22 cells with varying concentrations of CBD (0.02, 0.1, 10, 20, 50, and 100 µm) for 4 h and measured *Dec2* expression levels. The results showed that increasing CBD concentrations produced progressively greater upregulation of *Dec2* expression and corresponding reductions in *c‐fos* levels. Notably, effective *Dec2* mRNA upregulation was observed at CBD concentrations as low as 0.1 µm, a dose that falls well within the therapeutic window typically achieved in human plasma following oral administration of CBD (Figure 7C).^[^ ^30^ ^]^ This finding suggests that CBD may exert its anticonvulsant effects through DEC2‐mediated mechanisms.

To validate whether the DEC2‐SCN2A axis is critical for CBD's anticonvulsant effects, we employed a lentiviral knockdown approach in HT22 cells. Following successful DEC2 knockdown with lenti‐DEC2 shRNA in HT22 cells, treatment with 20 µm CBD failed to induce DEC2 upregulation at both mRNA and protein levels. Importantly, the characteristic CBD‐induced downregulation of SCN2A expression was also abolished in DEC2 knockdown cells (Figure 7D,E), demonstrating that intact DEC2 expression is required for CBD to modulate SCN2A levels. These results provide compelling evidence that the DEC2‐SCN2A axis serves as a critical pathway for CBD's molecular actions. We next sought to validate these findings in vivo by administering CBD (100 mg kg^−1^) to mice via intraperitoneal injection (Figure 7F). Consistent with our cellular observations, CBD treatment significantly increased hippocampal DEC2 expression while decreasing SCN2A levels at both protein and mRNA levels (Figure 7G,H), confirming that the DEC2‐SCN2A regulatory axis responds similarly to CBD treatment in both in vitro and in vivo systems.

Considering the interaction between DEC2 and MYOD1, we explored whether this interaction participates in CBD's mechanism of action. Using lentiviral delivery of MYOD1‐specific shRNA (LVX‐MYOD1 shRNA) in HT22 cells, we found that MYOD1 knockdown did not alter the ability of CBD to upregulate DEC2 expression or downregulate SCN2A expression (Figure 7I–L). Furthermore, co‐IP experiments in HEK293T cells co‐expressing DEC2‐HA and MYOD1‐FLAG revealed that CBD treatment did not alter the physical interaction between these two proteins (Figure 7M; Figure S7, Supporting Information). These results collectively indicate that the DEC2‐MYOD1 interaction is dispensable for CBD's modulation of the DEC2‐SCN2A pathway. In conclusion, our findings establish that CBD exerts its anticonvulsant effects through specific modulation of the DEC2‐SCN2A regulatory axis, leading to suppression of excessive neuronal activity. These results not only identify a novel molecular pathway for epilepsy treatment but also provide important insights into the sustained cellular and molecular mechanisms underlying CBD's therapeutic effects on neuronal function.

## 3 Discussion

**Figure 8:**
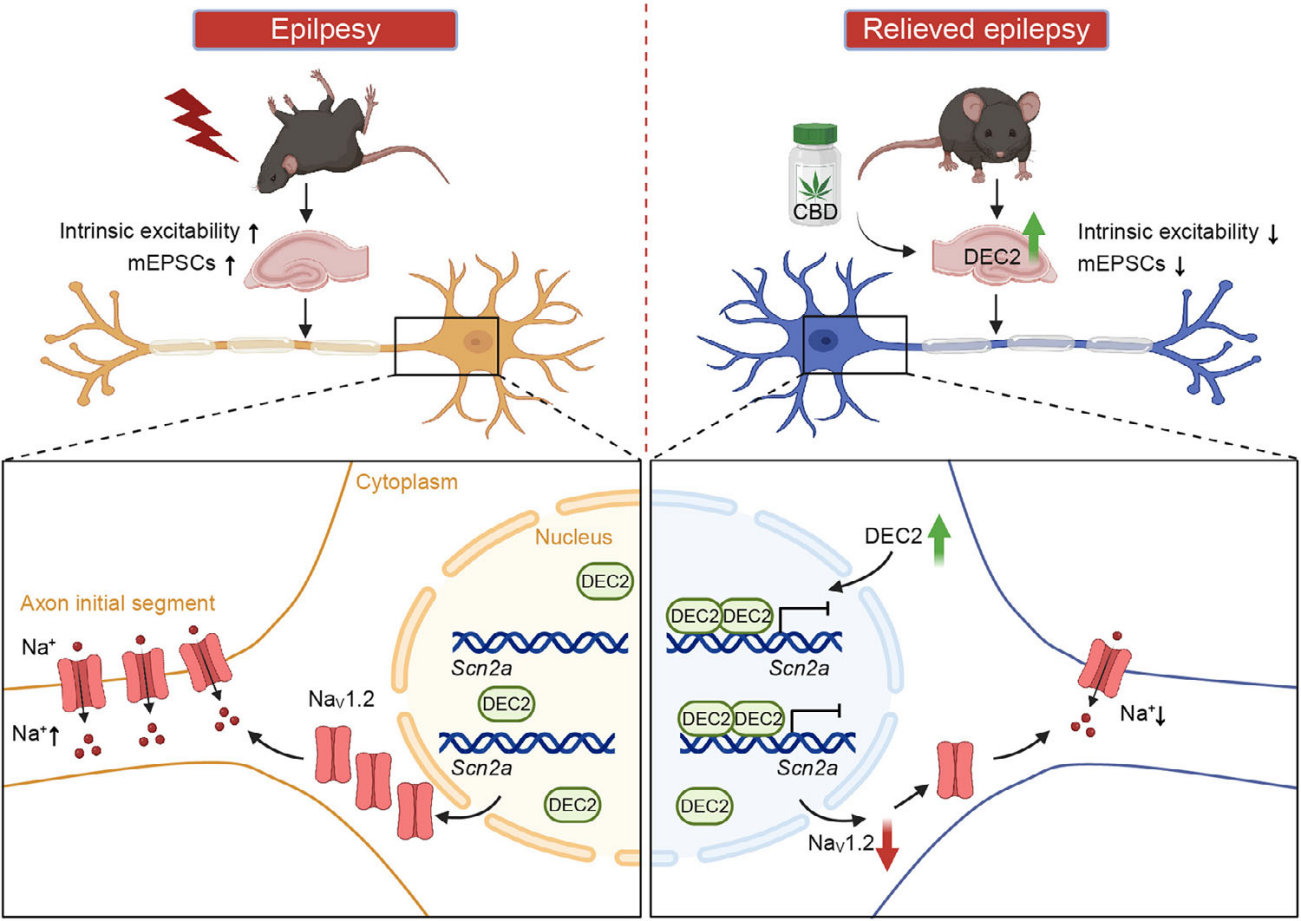
Proposed mechanism for DEC2‐mediated regulation of Na_V_1.2 channels and neuronal plasticity in epilepsy. In response to epileptic seizures, DEC2 expression is upregulated and acts as a transcriptional repressor of the *Scn2a* gene. DEC2 primarily regulates *Scn2a* by directly binding to its promoter, leading to reduced expression of membrane Na_V_1.2 channels. This downregulation decreases intrinsic excitability and synaptic transmission, which ultimately lowers seizure susceptibility and attenuates epileptogenesis. Furthermore, the DEC2‐SCN2A axis is modulated by CBD. CBD treatment enhances DEC2 expression, amplifying its repression of *Scn2a* transcription and contributing to its anticonvulsant effects.

In our study, we innovatively identify DEC2 as a novel modulator of neuronal plasticity in the central nervous system. DEC2 upregulation following seizure activity establishes a neuroprotective mechanism by directly repressing *Scn2a* transcription through binding to the *Scn2a* promoter. This transcriptional repression represents a previously unrecognized endogenous defense mechanism against epileptogenesis. Importantly, we demonstrate that CBD pharmacologically enhances this pathway by elevating DEC2 expression and reinforcing its inhibitory effects on *Scn2a* transcription, thereby contributing to its anticonvulsant effects (**Figure** **8**). Given that Na_V_1.2 has been implicated in various neurologic and psychiatric brain disorders, such as epilepsy, intellectual disability, and autism spectrum disorder, our study suggests the DEC2‐SCN2A axis may play a pivotal role in both physiological and pathological processes within the brain.

Neurons require plasticity to adapt to perturbations during development and learning, which involves dynamically adjusting their intrinsic excitability or synaptic function in response to the environment. Both these processes rely on intricate molecular mechanisms, including the activity‐dependent modulation of ion channels distributed across various neuronal compartments.^[^ ^1^, ^32^ ^]^ For instance, voltage‐gated sodium (Na_V_) and potassium KCNQ channels in the axon, as well as voltage‐gated potassium K_V_4 and hyperpolarization‐activated cyclic nucleotide‐gated (HCN) channels in the dendrites, are essential for the normal propagation of APs and the induction of LTP.^[^ ^33^ ^]^ The expression and function of these ion channels are regulated by gene transcription within neurons, and disruptions in this process can lead to epileptic seizures.^[^ ^34^ ^]^ Within this framework, the regulation of SCN2A is of particular interest.

While previous studies have identified only a limited number of transcription factors that suppress SCN2A expression, including RE1‐silencing transcription factor,^[^ ^35^ ^]^ histone H3K4 demethylase,^[^ ^36^ ^]^ and forkhead box D3.^[^ ^37^ ^]^ In this study, we unveil a previously unrecognized mechanism by which DEC2 acts as a novel and potent regulator of SCN2A. Through comprehensive AAV‐mediated targeted knockdown and overexpression experiments, we demonstrate that DEC2 knockdown elevates SCN2A expression in both mouse brain tissue and primary cortical neurons, whereas DEC2 overexpression results in decreased SCN2A levels (Figure 4E–L), establishing DEC2 as a key modulator of SCN2A expression. At the molecular level, our ChIP assays reveal DEC2's direct binding to two distinct E‐box elements within the *Scn2a* promoter, including the canonical DEC2 binding motif E‐box 1 (CACGTG) and E‐box 2 (CAGCTG), a site typically recognized by bHLH activator proteins such as MYOD1 (Figure 4O‐Q). This binding pattern initially raised the possibility of both direct and indirect regulatory mechanisms. However, subsequent luciferase reporter assays with site‐directed mutagenesis confirm that both E‐box elements are essential for full transcriptional repression (Figure 5A–C), though this system does not distinguish between direct and indirect modes of regulation. Crucially, functional analyses using DEC2 domain deletion mutants provide compelling evidence that direct DNA binding is the predominant mechanism of *Scn2a* regulation under physiological conditions. Deletion of the basic DNA‐binding domain (Δb) completely abolished DEC2's repressive activity, whereas deletion of the HLH dimerization domain (ΔHLH) had little effect on its function (Figure 5E–G). Collectively, these findings undoubtedly expand the transcriptional regulatory network of SCN2A.

Transcription factors emerge as promising candidates for modulating neuronal plasticity, as they can regulate multiple genes encoding various channels, transporters, proteins involved in both presynaptic and postsynaptic functions.^[^ ^7a^ ^]^ Among these, the bHLH family has emerged as important regulators of neuronal function. As a member of the bHLH‐Orange (bHLH‐O) subclass, DEC2 contains the characteristic bHLH domain and an adjacent Orange domain,^[^ ^38^ ^]^ enabling it to function primarily as a transcriptional repressor through either homodimer binding to E‐box elements (CANNTG) or heterodimer formation with other bHLH proteins.^[^ ^39^ ^]^ The bHLH‐O proteins play critical roles in neurogenesis and neural cell differentiation,^[^ ^40^ ^]^ and growing evidence implicates them in various neurological disorders,^[^ ^41^ ^]^ such as epilepsy.^[^ ^15^, ^42^ ^]^ However, the precise molecular mechanisms underlying these roles remain to be fully elucidated. In our study, we have established DEC2 as a pivotal regulator of epileptogenesis. Our genetic manipulation studies employing AAV‐mediated approaches reveal that DEC2 regulates seizure susceptibility, with knockdown exacerbating and overexpression attenuating epileptic phenotypes (Figure 2A–M). Through long‐term EEG‐video in a chronic epilepsy model, we demonstrate that DEC2 knockdown accelerates epileptogenesis, as evidenced by reduced latency to spontaneous recurrent seizures, increased seizure frequency and duration, and higher mortality rates (Figure 2N–S). These findings collectively position DEC2 as an endogenous brake on epileptogenesis, capable of modulating both seizure severity and disease‐associated mortality. At the cellular level, we demonstrate that DEC2 exerts profound effects on intrinsic and synaptic neuronal plasticity. Electrophysiological analyses reveal that DEC2 knockdown increases hippocampal neuronal intrinsic excitability and excitatory synaptic transmission, whereas overexpression produces the opposite effects (Figure 3; Figure S3, Supporting Information). Combined immunofluorescence staining and single‐cell RNA sequencing analyses of mouse hippocampal tissues confirm ubiquitous DEC2 expression across multiple cell populations (Figure 1K,L). While expressed in both excitatory and inhibitory neurons, DEC2's regulation of SCN2A occurs predominantly in glutamatergic circuits, consistent with the natural absence of SCN2A expression in GABAergic interneurons. This cellular specificity is further supported by our finding that DEC2 modulation does not alter inhibitory synaptic activity in GABAergic interneurons (Figure S4A–E, Supporting Information). These findings establish DEC2 as a crucial regulator of neuronal plasticity and epileptogenesis, significantly advancing our understanding of bHLH‐O protein function in the CNS disorders. While our neuronal overexpression studies provide compelling evidence for DEC2's effects in ameliorating hyperexcitability, future investigations using cell‐type‐specific manipulations (e.g., Cre‐loxP systems) will be valuable to elucidate DEC2's potential roles in glial cells and further dissect its neuron‐glia interactions in epileptogenesis.

Our transcriptomic and mechanistic studies collectively establish DEC2 as a master regulator of neuronal network homeostasis, with its influence extending far beyond the DEC2‐SCN2A axis. The RNA‐seq analysis of DEC2 knockdown mice reveals profound dysregulation across multiple functionally interconnected pathways that coordinately regulate neuronal integrity and network stability. Most notably, we observed coordinated changes in: 1) cell adhesion molecules (PCDH family, ITGB1) and extracellular matrix components (COL family, MMPs), particularly the striking downregulation of synaptic protocadherins (PCDHGA6‐9, PCDHGB6‐7) that normally stabilize specific neuronal connections; 2) synaptic transmission (SHANK3, GRIN3A) and differentiation factors (MYT1L, MECP2) that control both structural and functional plasticity; and 3) activity‐dependent signaling pathways (WNT, BMP, NRAS) and immediate early genes (FOS, FOSB) that mediate neuronal responses to stimulation. Given that epilepsy progression involves dynamic molecular changes, our findings indicate that DEC2 may act as a key modulator of epilepsy‐associated neuronal remodeling. The convergence of these pathways with DEC2's regulation of SCN2A suggests a multi‐layered mechanism of neuronal homeostasis: while direct SCN2A repression provides rapid control of intrinsic excitability, the parallel regulation of synaptic architecture (via PCDHs), postsynaptic organization (via SHANK3), and activity‐dependent signaling creates a complementary system for maintaining network stability. Notably, many affected genes (MECP2,^[^ ^43^ ^]^ SHANK3,^[^ ^44^ ^]^ and GRIN3A^[^ ^45^ ^]^) are themselves epilepsy‐associated, positioning DEC2 at a nodal point where it coordinately regulates multiple aspects of excitability (Figure 4A–C; and Table S5, Supporting Information). As the first comprehensive analysis of DEC2's transcriptional targets in neurons, these findings provide foundational insights into how this understudied transcription factor orchestrates neuronal function.

Despite the availability of over 25 licensed antiseizure drugs worldwide for symptomatic treatment of epileptic seizures, approximately one‐third of epilepsy patients continue to experience continuing seizures, a condition termed “drug‐resistant epilepsy”.^[^ ^46^ ^]^ CBD, the major active component of medical cannabis, has emerged as a promising therapeutic candidate for managing intractable epilepsy in both pediatric and adult populations with treatment‐ refractory epilepsies.^[^ ^11^, ^47^ ^]^ Preclinical investigations have extensively documented CBD's anticonvulsant efficacy, which is mediated through its interactions with diverse molecular targets including endocannabinoid receptors, transient receptor potential of vanilloid type‐1 channels, G protein‐coupled receptor 55, GABA receptors, 5‐hydroxytryptamine 1A serotonin receptors, adenosine pathway, and sodium and T‐type calcium channels.^[^ ^11^, ^47^, ^48^ ^]^ Our study uncovers a previously unreported mechanism underlying CBD's antiepileptic action, specifically through DEC2 upregulation and subsequent modulation of the DEC2‐SCN2A regulatory axis. Importantly, our in vivo experiments validate that CBD treatment induces consistent DEC2 elevation and SCN2A suppression in the mouse hippocampus (Figure 7F–H). The hippocampal strongly implicated in temporal lobe epilepsy pathogenesis. Notably, the dose CBD treatment used here (100 mg kg^−1^) align with regimens previously shown to reduce seizure frequency in murine models.^[^ ^49^ ^]^ Furthermore, our mechanistic studies reveal that CBD‐mediated DEC2 upregulation operates through MYOD1‐independent pathways, evidenced by both the maintained effects in MYOD1 knockdown conditions and the absence of CBD‐induced changes in DEC2‐MYOD1 binding affinity in co‐IP experiments (Figure 7I–M). Although the precise upstream pathways driving CBD‐induced DEC2 expression require further elucidation, our findings suggest that enhancement of DEC2‐mediated SCN2A repression may represent an innovative therapeutic strategy for drug‐resistant epilepsy.

## 4 Conclusion

In summary, our study not only identifies DEC2 as a pivotal regulator of activity‐dependent neuronal plasticity and epilepsy progression but also provides a comprehensive understanding of its regulatory mechanisms and therapeutic potential. A notable increase in DEC2 expression has been observed at both the protein and mRNA levels in response to heightened neuronal activity. This upregulation is associated with a reduction in intrinsic excitability and excitatory synaptic activity of hippocampal cells, as well as a concurrent suppression of seizure susceptibility. Further investigation revealed that DEC2 functions as a transcriptional repressor that downregulates Na_V_1.2 channel expression primarily through direct DNA binding, with a limited contribution from indirect protein‐protein interactions. This mechanism facilitates the downregulation of Na_V_1.2 channels at the neuronal membrane, providing an exclusive molecular pathway underlying Na_V_1.2 channel modulation.

## 5 Experimental Section

### Materials

pGL3‐basic luciferase reporter vector, pRL‐TK vector, pcDNA3.1‐*Dec2*‐3 × FLAG, pcDNA3.1‐*Myod1*‐HA were purchased from YouBio (Hunan, China). pLVX‐*Myod1* shRNA plasmid was purchased from Tsingke Biotechnology (Beijing, China). PB2A‐GFP‐*Dec2*, pLenti‐*Dec2*‐3 × FLAG, pMD2.G, VSV‐G (Addgene Catalog #12 259), psPAX2 (Addgene Catalog #12 260) were obtained from Addgene (America).

Commercial antibodies used were: rabbit anti‐DEC2 (Abcam, ab175544, 1:5000 for western blot, 1:100 for immunofluorescence staining), mouse anti‐SCN2A (Alomone labs, ASC‐002, 1:400 for western blot), mouse anti‐MYOD1 (Santa Cruz Biotechnology, sc‐377460, 1:1000 for western blot), mouse anti‐GAPDH (Abcam, ab8245, 1:5000 for western blot), anti‐FLAG (Abbkine, ABT2010), anti‐IgG (Biodragon, BF01006), mouse anti‐NeuN (Millipore, MAB377, 1:800 for immunofluorescence staining), goat anti‐GFAP (Abcam, ab53554, 1:300 for immunofluorescence staining). Mouse anti‐CaMKIIα (Santa Cruz Biotechnology, sc‐13141, 1:500 for immunofluorescence staining) and mouse anti‐GAD67 (Millipore, MAB5406, 1:500 for immunofluorescence staining) were obtained from Prof. Yong Zhang at Peking University.

Protein A/G beads were from GE Healthcare Biosciences, protease inhibitor mixture cocktail was from Roche Applied Science. TTX was from Absin Bioscience. Bicuculline, CGP 55 845, CNQX, and DL‐AP5 were from Abcam. TSA was from Adipogen. CBD was provided by Hanyi Bio‐technology Company Ltd (Beijing). Carbamazepine, sodium valproate, levetiracetam, oxcarbazepine, topiramate, and lamotrigine were from Innochem. All other reagents were purchased from Sigma‐Aldrich.

### Human Specimens

Patients (n = 16) with medically intractable TLE underwent phased presurgical assessment at Shengjing Hospital affiliated to China Medical University. Epilepsy diagnosis (including types and localization) was determined by clinical history, imaging examination (including MRI and/or PET), EEG (including scalp and/or intracranial EEG), and psychological assessment. In those selected for surgery, the temporal lobe was resected according to standard procedures between March 2013 and October 2013. The study using clinical samples, which include 8 normal tissues and 8 epileptogenic tissues, was approved by the Ethics Committee of Shengjing Hospital affiliated to China Medical University (Table S1, Supporting Information). Tissues were frozen in liquid nitrogen immediately after surgical removal and maintained at −80 °C until protein extraction. Informed consent was obtained from all subjects or their relatives.

### Animals

Male C57BL/6 mice (6–8 weeks) were purchased from Charles River Laboratories (Beijing). *Scn8a* (C3HeB/FeJ background) heterozygote mice were obtained from Prof. Yousheng Shu at Fudan University. *Scn8a*‐KO mice were reproduced by intercrossing the heterozygous mice and validated in this laboratory. PCR genotyping information for *Scn8a* was as follows: a 164 bp fragment was indicative of wild‐type allele, and an 82 bp fragment was indicative of null allele. Heterozygotes showed the fragments of both 164 and 82 bp. (Wild type forward: 5′‐TCAGGAGCAAGGTTCTAGGC‐3′; Mutant forward: 5′‐TACCAAAAGTCCCCATACCC‐3′; Common reverse: 5′‐AGGAGTGGCGCTAAATCTGA‐3′). All mice were maintained under a 12 h light/dark cycle (at controlled room temperature of 22–25 °C and a relative humidity of 40–60%), and with free access to food and water. All experiments were performed according to the guidelines of the Animal Care and Use Committee of Peking University. Every effort was made to minimize animal suffering and to reduce the number of animals used. The experiments were blind to viral treatment or genotype during behavioral testing.

### Weighted Gene Co‐Expression Network Analysis

The R package WGCNA was employed to construct a weighted co‐expression network for the identification of modules and hub genes associated with TLE. Gene expression profiles of GSE47752 were downloaded from the GEO database. Separate analyses were conducted for the rat KA, Pilo, kindling, and SSSE models. After filtering out genes with low variability or missing values, 31 099 genes were retained to construct the co‐expression matrix in these models. The data were filtered by removing genes with missing values and then used to calculate Pearson correlation between all gene pairs. The pickSoftThreshold function in WGCNA was utilized to determine the appropriate soft‐thresholding power (β) that met the criterion of approximating a scale‐free topology of the network. Subsequently, using this approach, adjacency matrices were constructed with soft‐thresholding powers of 3 (KA), 15 (Pilo), 4 (Kindling), and 14 (SSSE) in this study. The resulting adjacency matrix was used to calculate topological overlap matrix (TO), which was further hierarchically clustered with (1‐TO) as a distance measure. Co‐expression modules were then determined using a dynamic tree‐cutting algorithm, with a minimum module size requirement of 30 genes. Modules with a module eigengene (ME) distance below 0.25 were merged. To assess module membership (MM), the Pearson correlation between each gene and its corresponding ME was calculated. Each gene was assigned to the module with the highest MM. The gene list of the modules of interest was extracted, and a scatterplot was generated for these genes. Additionally, gene significance (GS) was computed, representing the Pearson correlation between each gene and the phenotype (1 for epilepsy, 0 for control).^[^ ^12^, ^20^ ^]^

### Kainic Acid‐Induced Status Epilepticus and Pentylenetetrazole‐Induced Seizures

Mice were injected with a single i.p. dose of KA (Sigma‐Aldrich) at 20 mg kg^−1[^ ^18^, ^33^ ^]^ or PTZ (Sigma‐Aldrich) at 80 mg kg^−1^ to induce class IV or higher, according to the modified Racine scale as described.^[^ ^19^ ^]^ These were terminated 90 min after onset using sodium pentobarbital (SP) (37 mg kg^−1^, Sigma‐Aldrich). Control groups were mice that had been treated with normal saline and SP. Following the administration of KA, mice were sacrificed for protein and RNA extraction at 1‐, 7‐, and 14‐ days post‐injection.

In the epilepsy susceptibility test, animals were tested for seizure behavior in the dark phase. Seizures were induced by intraperitoneal administration of 20 mg kg^−1^ KA and video‐recorded for 90 min. To assess epilepsy susceptibility, seizures were rated using a modified Racine scale^[^ ^19^ ^]^: 1) immobility followed by facial clonus; 2) masticatory movements and head nodding; 3) continuous body tremor or wet‐dog shakes, unilateral forelimb clonus; 4) bilateral forelimb clonus; 5) rearing and falling, generalized tonic‐clonic convulsions or hyperactivity/jumping behavior.

### Chronic Epilepsy Induction and EEG Recordings

Male C57BL/6 mice were anesthetized using 3% isoflurane (induction) and maintained at 1–2% isoflurane during surgery while secured in a stereotaxic frame (RWD Ltd, China). Animals were randomly divided into two experimental groups receiving either control shRNA or AAV‐DEC2 shRNA‐RFP through bilateral hippocampal DG injections. For chronic EEG monitoring, four subdural screw electrodes were surgically implanted at stereotaxically defined positions over the left and right dorsal hippocampus and frontal cortex. These electrodes were connected to a 6‐pin pedestal connector that was centered over the skull and secured with dental cement.

After allowing 12–14 days for postoperative recovery and AAV transgene expression, baseline EEG activity was monitored for two consecutive days to verify the absence of spontaneous seizure activity. Chronic epilepsy was subsequently induced on day 14 post‐injection through intraperitoneal administration of KA (25 mg kg^−1^). Status epilepticus was pharmacologically terminated after 2 h using SP (37 mg kg^−1^, i.p.). Continuous EEG‐video monitoring was performed daily for 28 days using a BIOPAC MP150 acquisition system (sampling rate: 500 Hz; bandpass filter: 1–100 Hz) synchronized with infrared video recording. Each daily recording session lasted 12 h to ensure comprehensive seizure detection.

All EEG recordings were analyzed offline using Sirenia Seizure Pro (v.1.8.4, Pinnacle Technology). Seizures were detected using an automated detection algorithm, followed by manual verification. Seizures were defined as rapid and rhythmic (>3 Hz) deflections in all EEG channels that lasted >10 s and were at least 3 standard deviations above the baseline root mean square amplitude. Seizures were confirmed convulsive if the video showed behaviors consistent with stages 3–5 on the Racine scale. The following parameters were assessed: 1) Time taken to reach first spontaneous seizure. 2) Mean seizures/day (number of seizure events per day). 3) Mean seizure duration (mean duration per seizure episode). 4) Mortality rate (percentage of animals that succumbed to epilepsy‐related complications).

### In Vivo CBD Treatment

CBD was prepared in soybean oil vehicle at a concentration of 100 mg kg^−1^. Male C57BL/6 mice were randomly allocated into two experimental groups: 1) CBD‐treated group receiving 100 mg kg^−1^ CBD or 2) vehicle control group receiving soybean oil alone. Animals received intraperitoneal injections twice at 12‐h intervals (0 and 12 h). At 24 h after the initial injection (12 h following the second dose), mice were deeply anesthetized and transcardially perfused with ice‐cold PBS. Hippocampal tissue was immediately dissected and processed for simultaneous protein and RNA extraction.

### Quantitative Real‐Time Reverse Transcription (RT)‐PCR Assay

Total RNA was isolated from cell and tissue samples with TRIzol reagents (Invitrogen, 15 596 018), and reversely transcribed to cDNA using the Reverse Transcription System (TransGen Biotech, AT301‐02) according to the manufacturer's instructions. Relative quantitation was determined using ABI fluorescence quantitative PCR instrument (QuantStudio 6) that measures real‐time SYBR green fluorescence and then calculated by means of the comparative Ct method (2^−ΔΔCt^) with the expression of β‐actin as an internal control. Primer sequences are summarized in Table S8 (Supporting Information).

### Western Blot Analysis

Protein samples were separated by SDS‐PAGE on 8% tris‐acetate gels and electro‐transferred to 0.45 µm nitrocellulose membranes (GE Healthcare, 10 600 003). After blocking, the membranes were incubated with the primary antibodies, followed by incubation with HRP‐conjugated secondary antibodies (Biodragon, BF03001 and BF03008). Immunoreactive bands were visualized using Western Blotting Luminal Reagent (Santa Cruz Biotechnology) according to the manufacturer's recommendation. The molecular weights were determined by using an appropriate pre‐stained protein standard (Biotides, WB1902; Thermo Scientific, 26 616 and 26 619). Quantitative analysis was determined by densitometry using ImageJ.

### Immunofluorescence Staining

Brain samples were fixed in 4% paraformaldehyde, and then transferred to a grade series of sucrose solution (20% and 30%). Cryostat coronal sections (20 µm) were blocked with 5% goat serum and 0.1% Triton X‐100 in PBS (vol/vol), and then incubated with primary antibody. After washing with PBS, sections were incubated with Alexa 488‐conjugated goat anti‐rabbit IgG (Abcam, ab150077), Alexa 594‐conjugated donkey anti‐goat IgG (Abcam, ab150132) and Alexa 647‐conjugated goat anti‐mouse IgG (Abcam, ab150115). Sections were washed, mounted, and imaged using Zeiss LSM510 confocal microscope (Jena, Germany) or Nikon fluorescent microscope (Tokyo, Japan).

### Virus Infection

AAVs carrying shRNA targeting mouse DEC2 (RFP vector) were from Vigene Biosciences (Shandong). AAVs carrying shRNA targeting mouse SCN2A (GFP vector) were from GeneChem (Shanghai). Lentiviruses carrying shRNA‐targeting mouse DEC2 lentiviral vectors (RFP vector, lenti‐DEC2 shRNA) were from GeneChem (Shanghai). The infection efficiency was confirmed by the expression of green or red fluorescent protein under microscopy. The DEC2 shRNA#1 sequence is: 5′‐AGAAAGCAGTAGTCTTGGAATTTCAAGAGAATTCCAAGACTACTGCTTTCTTTTTTT‐3′. The DEC2 shRNA#2 sequence is: 5′‐GGACGAAGGAATCCCTCATTTCAAGAAAATGAGGGATTCCTTCGTCCTTTTTT‐3′. The DEC2 shRNA#3 sequence is: 5′‐GCAGCATCAGAAGATAATTTCAAGAGAATTATCTTCTGATGCTGCTTTTTT‐3′. The SCN2A shRNA sequence is: 5′‐ATCAAATCCCTCCGAACATTA‐3′. The non‐silencing shRNA sequence is: 5′‐TTCTCCGAACGTGTCACGT‐3′. The DEC2 siRNA sequence is: 5′‐GAAAGCAGTAGTCTTGGAATT‐3′. AAVs carrying *Dec2* full length (GFP vector) were from Vigene Biosciences (Shandong). AAVs carrying *Dec2*‐3xFLAG (EGFP vector) were from OBiO Technology (Shanghai). Three weeks after AAV injection, tissues were collected and either protein or RNA was extracted for western blot or RT‐PCR, respectively.

### Stereotaxically Guided AAVs Injection

Mice were anesthetized with the inhalation anesthetic isoflurane and positioned in thestereotaxic apparatus (RWD Ltd, China). After craniotomy, mice were bilaterally injected with 0.5 µL viruses into the hippocampal DG (coordinates, bregma: anterior/posterior: −2.0 mm, medial/lateral: ±1.3 mm, dorsal/ventral: −2.1 mm) or CA1 regions (coordinates, bregma: anterior/posterior: −2.5 mm, medial/lateral: ±2.2 mm, dorsal/ventral: −1.8 mm). After injection, the needle was maintained in the place for an additional 10 min to facilitate the diffusion of the virus and then slowly withdrawn. Mice receiving virus injections were returned to their home cages to recover for 2–4 weeks before they were subjected to electrophysiological recordings or behavioral tests.

### RNA Sequencing

Total mRNA was extracted from mice hippocampus tissues infected with control shRNA and AAV‐DEC2 shRNA‐RFP. High‐throughput RNA‐seq was performed by Illumina NovaSeq 6000 (Illumina, San Diego, CA) at CapitalBio Corporation (Beijing, China). The raw sequencing data were aligned to the mouse reference genome (GRCm38, mm10). For RNA‐seq differential gene expression analysis, a significance threshold of *p* < 0.05 was applied to identify DEGs. GO analysis was subsequently performed on these FDR‐corrected gene sets using DAVID tools with additional FDR correction (*q*‐value < 0.05). The complete GO enrichment results and associated gene lists are provided in Tables S5 (Supporting Information). The raw gene expression abundance data from the sequencing results are presented in Table S10 (Supporting Information).

### Brain Slice Preparation and Whole‐Cell Current‐Clamp Recordings

Horizontal slices were obtained from 8–10 weeks old male C57BL/6 mice infected with AAVs. In brief, animals were anesthetized and decapitated into an ice‐cold slicing solution containing (in mm): 110 choline chloride, 2.5 KCl, 1.25 NaH_2_PO_4_, 25 NaHCO_3_, 0.5 CaCl_2_, 7 MgCl_2_, 10 glucose. Brain slices (300 µm thick) were prepared in ice‐cold slicing solution with a vibratome (Leica VT1200S, Germany). Slices containing hippocampus were incubated at 37 °C for 15 min in an “external solution” containing (in mm): 125 NaCl, 2.5 KCl, 1.25 NaH_2_PO_4_, 25 NaHCO_3_, 2 CaCl_2_, 2 MgCl_2_, 10 glucose (315 mOsm, PH 7.4), and then stored at room temperature for 1 h before use. All solutions were bubbled continuously with 95% O_2_ and 5% CO_2_.

Data were collected with a Multiclamp 700B amplifier (Molecular Devices), filtered at 10 kHz and sampled at 50 kHz. Whole‐cell recordings were obtained with patch pipettes (3–6 MΩ) filled with different internal solutions according to experiments. Series resistance was in the order of 10–30 MΩ and was ≈60–80% compensated. Recordings were discarded if the series resistance increased by more than 20% during recordings. Data were acquired and analyzed using pClamp 10.4 (Molecular Devices).

For whole‐cell current‐clamp recordings, the external solution (unless otherwise noted) was supplemented with 50 µm APV, 10 µm CNQX, 10 µm bicuculline, 1 µm CGP 55 845 and internal pipette solution containing (in mm): 118 KMeSO_4_,15 KCl, 10 HEPES, 2 MgCl_2_, 0.2 EGTA, 4 Na_2_ATP, 0.3 Tris‐GTP, 14 Tris‐phosphocreatinin (295‐300 mOsm, PH 7.3). For mEPSCs recordings, the “external solution” was supplemented with 0.5 µm TTX, 10 µm bicuculline and 1 µm CGP 55 845. For mIPSCs recordings, the “external solution” was supplemented with 0.5 µm TTX, 50 µm APV, 10 µm CNQX and the internal solution containing (in mm): 122 CsCl, 1 CaCl_2_, 5 MgCl_2_, 10 EGTA, 10 HEPES, 4 Na_2_ATP, 0.3 TrisGTP, 14 Tris‐phosphocreatine (295‐300 mOsm, PH 7.3).

### Behavioral Analysis

In the open field test, animals were allowed to explore freely for 5 min in the open field area (60 cm × 60 cm × 60 cm), the travel distance and the time spent in the delineated center zone were videotaped and measured by SMART software (v2.0) to reflect exploratory and anxiety‐related behavior. The field was cleaned by 75% ethanol between tests.

In the elevated plus maze, a plus‐shaped platform was used at 50 cm above the floor with two open arms (30 cm × 5 cm) and two closed arms (30 cm × 5 cm × 40 cm) on opposing sides of a central square platform (5 cm × 5 cm). The overall illuminations of the 4 arms were kept equal, at 100–200 lux. Each animal was gently placed in the center platform facing an open arm and was videotaped for 5 min. The total time and entries in the open arms (30 cm × 5 cm) were measured using the software SMART. Animals that fell from the maze were removed from the experiment.

In the sucrose‐preference test, animals were habituated to drinking water from two bottles for 2 days. In the sucrose preference test, two pre‐weighed bottles [one containing tap water and the other containing a 1% (w/v) sucrose solution] were presented to each animal for 24 h. The position of the water and sucrose bottles (left or right) was switched 24 h later. The bottles were weighed again, and the weight difference represented the animal's intake from each bottle. The sum of water plus sucrose intake was defined as the total intake, and sucrose preference was expressed as the percentage of sucrose intake relative to the total intake [preference = (sucrose intake/total intake) × 100%].

In the tail suspension test, animals were hung 15 cm above the floor by the tip of the tail (1 cm) and were adhered to an aluminum bar. The total test procedure of mouse immobility time was counted during a test period of 6 min (2 min of adaptation time and 4 min of recording). A mouse was considered immobile when it was passively suspended and completely motionless. Immobility time was defined as the immobile state in the last 4 min of the total 6 min.

### Isolation of Mice Primary Cortical Neurons

To culture primary neurons, cortex from postnatal day 0–1 C57BL/6 and C3HeB/FeJ newborn pups were dissected in ice‐cold HBSS (Gibco, 14 025 092) containing 1% penicillin/streptomycin (P/S) (Gibco, 15 140 122), and then digested in 0.25% Trypsin (Lonza, 17–161E) at 37 °C for 20 min. Digestion was halted by adding two volumes of DMEM (Gibco, C11995500BT) supplemented with 10% fetal bovine serum (FBS) (ExCell Bio, FSP500). The cell suspension was pelleted, and the cells were then plated on culture dishes pre‐coated with poly‐D‐lysine (PDL, Sigma‐Aldrich, COR3670‐100EA) and grown for 4 h in plating medium. Thirty‐five millimeters PDL‐coated dishes were used for cell electrophysiology experiments, 60 mm PDL‐coated dishes for western blot and RT‐PCR or analyses, and PDL‐coated 150 mm dishes for ChIP assays. Then, primary cultures were completely replaced with Neurobasal medium (Gibco, 21 103 049) containing 1% Glutamax (Gibco, 35 050 079), 1% P/S, and 2% B‐27 (Gibco, 17 504 044) and grown for 6–9 days with half of the media replaced every three days.

### Preparation of Lentiviruses

For lentiviral production to infect mouse primary neurons and the HT22 cells, HEK293T/17 cells were cultured in 100 mm diameter dishes with 10% (v/v) FBS‐containing DMEM medium. After 24 h, at 80% cell confluency, cells were transfected with polyethyleneimine (PEI) with the following plasmid combinations (total 10 µg per dish): (1) For DEC2 constructs: 6 µg pLenti‐*Dec2*‐3 × FLAG (mouse) (or deletions pLenti‐*Dec2* Δb‐3 × FLAG/pLenti‐*Dec2*‐ΔHLH‐3 × FLAG) + 2 µg VSV‐G + 2 µg psPAX2; (2) For MYOD1 knockdown: 6 µg pLVX‐*Myod1* shRNA (mouse) + 2 µg VSV‐G + 2 µg psPAX2. The primers used for generating DEC2 deletions from pLenti‐*Dec2*‐3 × FLAG plasmid were as follows: DEC2‐Δb forward: 5′‐GAGCTTGAAGCGAGACGATACCAAGGACCGAATTAATGAATGCATT‐3′; DEC2‐Δb reverse: 5′‐AATGCATTCATTAATTCGGTCCTTGGTATCGTCTCGCTTCAA‐3′. DEC2‐ΔHLH forward: 5′‐TTAATAGAAAAGAAGAGACGAGCCTTAACTGAGCAGCAGCAT‐3′; DEC2‐ΔHLH reverse: 5′‐ATGCTGCTGCTCAGTTAAGGCTCGTCTCTTCTTTTCTATTAA‐3′. After 6 h post‐transfection, the media were exchanged with DMEM medium containing 10% (v/v) FBS. At 72 h post‐transfection, the supernatant was harvested and concentrated using the Lenti‐X concentrator reagent (Takara, 631 231), following the manufacturer's instructions. The concentrated lentiviral particles were subsequently subjected to centrifugation at 14 000 rpm for 1 h and resuspended in PBS.

### Whole‐Cell Voltage‐Clamp Recordings

The lenti‐DEC2 shRNA was used to infect primary neurons on day 3 at an optimal multiplicity of infection (MOI) of 10. After an 8 h incubation, 50% of the medium was replaced with fresh neurobasal medium. Subsequently, half of the culture medium was changed to maintain optimal conditions every 3 days. The plasmids expressing GFP‐*Dec2* were transfected into primary neurons using lipofectamine 3000 (ThermoFisher Scientific, L3000015) at day 4 following the standard protocol. To isolate Na_V_1.2 current, the recording was performed between day 6–7 days. After infection or transfection, cells were placed on a glass chamber with extracellular solution containing (in mm): 140 NaCl, 3 KCl, 10 HEPES, 10 D‐glucose, 1 MgCl_2_, 1 CaCl_2_ (pH 7.3, adjusted with NaOH; 310 mOsm). Recordings were made from isolated, GFP or RFP‐positive cells using 1.5–2.5 MΩ fire polished pipettes (Sutter Instruments) filled with standard internal solution composed of (in mm) 140 CsF, 10 HEPES, 1 EGTA, 10 NaCl (pH 7.3, adjusted with CsOH; 300 mOsm).

Currents were recorded using a HEKA EPC‐10 patch‐clamp amplifier (HEKA Elektronik, Germany), low pass filtered at 5 kHz and sampled at 20 kHz. The cells with series resistance in the range of 2–6 MΩ were accepted for the further investigations, and the series resistance was compensated by ≈70–90%. Recordings were discarded if the series resistance was increased to more than 6 MΩ during recording. The data were acquired by PatchMaster program (HEKA Elektronik). All experiments were performed at room temperature.

To assess the current amplitude of Na_V_1.2, neurons were held at −120 mV and the inward sodium currents were elicited by a 50 ms step to −10 mV. To characterize the voltage dependence of activation of Na_V_1.2, cells were held at −120 mV and then a series of 100 ms test pulses from −80 to +40 mV (5 mV increments) were applied. Conductance‐voltage (*G*‐*V*) relationships were generated using a Boltzmann equation.

(1) $$I/\mathrm{Imax}={\left(1+\exp\left[\left(V_{m-}V_{1/2}\right)/k\right]\right)}^{-1}$$

(2) $$G/G_{\max}={\left(1+\exp\left[\left(V_{m-}V_{1/2}\right)/k\right]\right)}^{-1}$$

(3) $$G=I/\left(V_{m-}E_{\mathrm{Na}}\right)$$

where *I* is the peak current, *G* is the conductance, *V_m_* is the test potential, *V_1/2_* is the half‐maximal activation potential, *E_Na_* is the equilibrium potential, and *k* is the slope factor.

### *Scn2a* Promoter Luciferase Constructs

Mouse genomic DNA was extracted using a purification kit (ThermoFisher Scientific, K0512), and then used as a template for PCR amplification. The 1.5 kb fragment of the *Scn2a* promoter region containing three E‐box elements was amplified using the following primers: Forward primer: 5′‐GAGCTCTTACGCGTGCTAGCGAACGTGCAAGGATTTTCTTGATGC‐3′. Reverse primer: 5′‐CGCAGATCTCGAGCCCGGCTTTTCATCCTGCTTCTTTAATCACTGTTTAGCTCCTCGC‐3′. The PCR amplified product was subsequently cloned into the NheI and XhoI restriction sites of the pGL3‐basic luciferase reporter vector to generate the wild‐type reporter construct (designated *Scn2a* pro‐luc). To generate specific mutations in the three E‐box elements of the *Scn2a* promoter, the following primer was designed and utilized: Forward_M1: 5′‐GCCACCCCTACCGGTTTATCTTAATGGTCATTGCTTTTTCCCCTCCTGTTTCTGTAGC‐3′; Reverse_M1: 5′‐TAAGATAAACCGGTAGGGGTGGCAATCACAGGACACTGAGCATCAAG‐3′. Forward_M2: 5′‐CTGGAAGACGCGTCCTTTGGGAAGTGCTCTAGCTGTTTTGCTTTGCATACT‐3′; Reverse_M2: 5′‐CCAAAGGACGCGTCTTCCAGCGCTGTGAGCCCTCATGC‐3′. Forward_M3: 5′‐ATCTGAACGCGTGTGCCTGAAAGGTGCTGGTCAACTTTAAAAATAAAAATTAATGAAA‐3′; Reverse_M3: 5′‐AGGCACACGCGTTCAGATTGCCCTGTCAGCATCACAGG‐3′.

### Transfection and Luciferase Assay

HEK293T cells were seeded in 96‐well plates at a density of 3.4 × 10⁴ cells well^−1^ and cultured for 24 h prior to transfection. Cells were co‐transfected using Lipofectamine 2000 (ThermoFisher Scientific, 11 668 019) according to the manufacturer's instructions, with each well receiving a total of 150 ng DNA consisting of either: 1) wild‐type *Scn2a* pro‐luc reporter construct or its E‐box mutant variants, 2) pcDNA3.1‐*Dec2*‐3 × FLAG plasmid, or 3) empty pcDNA3.1 vector (Invitrogen). The total DNA concentration was adjusted to 150 ng per well with empty pcDNA3.1 vector. For the internal standard, 3 ng of pRL‐TK vector (YouBio) was transfected into each well. After 48 h of transfection, cells were lysed and dual‐luciferase activity was measured using the provided reagents from the Dual‐Lumi Luciferase Reporter Gene Assay Kit (Beyotime, RG088S). Luminescence was measured with a luminometer (LB960, Berthold, Germany). For TSA treatment, cells were treated with either 100 nm TSA or a vehicle control. Twenty‐four hours after TSA treatment, the cells were collected for luciferase assay.

### Co‐Immunoprecipitation (Co‐IP) Assay

HEK293T cells cultured in 100 mm dishes were co‐transfected with 6 µg each of pcDNA3.1‐*Dec2*‐HA and pcDNA3.1‐*Myod1*‐3 × FLAG plasmids using Lipofectamine 2000. After 48 h post‐transfection, cells were lysed in RIPA lysis (APPLYGEN, C1053) supplemented with protease inhibitor cocktail for 30 min at 4 °C. The cell lysates were then centrifuged at 15 000 g for 20 min at 4 °C to remove cellular debris and the supernatant was incubated with antibody for 12 h at 4 °C with constant rotation. Protein G Dynabeads (ThermoFisher Scientific, 10009D) were added and incubation continued overnight at 4 °C. Beads were collected using a DynaMag and washed three times with RIPA lysis. The remaining proteins were eluted from the beads by resuspending the beads in 1 × SDS‐PAGE loading buffer and incubating for 10 min at 95 °C. The resultant materials were then subjected to western blot analysis. Input samples were loaded at 5% of the total lysate volume. For CBD treatment experiments, CBD was added 12 h prior to protein extraction at a final concentration of 20 µm.

### ChIP and qChIP

For the ChIP experiments of mouse DEC2 overexpression in primary neurons, lenti‐DEC2‐3 × FLAG was introduced into primary neurons using the same lentiviral transduction method described above. The neurons in the 150 mm dishes can be harvested and used for ChIP experiments between day 7–9. For in vivo ChIP experiments, mice were stereotaxically injected with AAV‐DEC2‐3 × FLAG bilaterally into the DG of hippocampus. Following a 4‐week period for viral expression and protein production, mice were deeply anesthetized and transcardially perfused with ice‐cold PBS, followed by immediate microdissection of DG regions.

ChIP experiments were performed according to the procedure described previously.^[^ ^18^ ^]^ Mice primary cortical neurons or SY5Y cells were fixed with 1% formaldehyde for 10 min at room temperature. The fixed cells were lysed in lysis buffer (1% SDS, 5 mm EDTA and 50 mm Tris‐HCl (pH 8.1), plus protease inhibitor cocktail). The lysates were then sonicated with 3 × 10 cycles (30 s on and off) (Bioruptor, Diagenode) to generate chromatin fragments of ≈300 bp in length. Cell debris was removed by centrifugation and the supernatant was collected. A dilution buffer (1% Triton X‐100, 2 mm EDTA, 150 mm NaCl, 20 mm Tris‐HCl (pH 8.1), plus protease inhibitor cocktail) was subsequently applied (1:10 ratio) and the resultant chromatin solution (aliquot 50 µL as the input) was then incubated with control or specific antibodies (3‐5 µg) for 12 h at 4 °C with constant rotation. Protein G Dynabeads (ThermoFisher Scientific, 10009D) (50 µL of 50% (vol/vol)) were added for incubation of another 3 h. Beads were collected by centrifugation at 800 g for 5 min at 4 °C. Beads were sequentially washed with the following buffers for 5 min at 4 °C: TSE I (0.1% SDS, 1% Triton X‐100, 2 mm EDTA, 150 mm NaCl, 20 mm Tris‐HCl (pH 8.1)); TSE II (0.1% SDS, 1% Triton X‐100, 2 mm EDTA, 500 mm NaCl, 20 mm Tris‐HCl (pH 8.1)); buffer III (0.25 m LiCl, 1% Nonidet P‐40, 1% sodium deoxycholate, 1 mm EDTA, and 10 mm Tris‐HCl (pH 8.1)); Tris‐EDTA buffer. The input and the precipitated DNA‐protein complex were de‐cross‐linked at 65 °C for 12 h in elution buffer (1% SDS, 5 mm EDTA, 50 mm NaCl, 0.1 mg mL^−1^ proteinase K, 20 mm Tris‐HCl (pH 8.1)), and DNA was purified using PCR purification kit (Qiagen). Quantification of the precipitated DNA fragments was performed with real‐time PCR using primers listed in Table S9 (Supporting Information).

### Statistics Analysis

For in vivo experiments, the animals were distributed into various treatment groups randomly. For in vitro experiments, the cells were evenly suspended and then randomly distributed in each well tested. All the data were represented as the mean ± SEM. The analysis of data was performed with GraphPad Prism 6 (GraphPad Software). Comparisons between two groups were made using an unpaired two‐tailed Student's t‐test. Comparisons among three or more groups were made using one‐ or two‐way ANOVA with Bonferroni's multiple‐comparisons test. A *p* value less than 0.05 was considered statistically significant. (*p < 0.05, **p < 0.01, ***p < 0.001, and n.s. no statistical significance). All experiments and analysis of data were performed in a blinded manner by investigators who were unaware of the experimental group or manipulation.

## Conflict of Interest

The authors declare no conflict of interest.

## Author Contributions

H.S. performed and analyzed western blot, RT‐PCR, RNA‐seq data, ChIP assays, luciferase assays, virus injection, immunofluorescence staining, patch‐clamp recordings and behavioral tests. Y.W. performed and analyzed WGCNA analysis, constructed the pLenti‐*Dec2*‐3 × FLAG vector, and assisted with western blot, immunofluorescence staining, and plasmid construction. L.W. assisted with western blot and co‐IP experiments. C.G. conducted chronic epilepsy EEG electrode implantation, recording, and data analysis. S.L. performed and analyzed whole‐cell voltage‐clamp recordings of Na_V_1.2 current. Y.R. assisted with preparing samples for western blot and co‐IP experiments. J.T. assisted with constructing DEC2 deletion plasmids, site‐directed mutagenesis‐related plasmids, and RT‐PCR. C.P. assisted with virus injection, single‐cell RNA‐seq and bulk RNA‐seq data analyses. Y.S. assisted with AAV virus injections. Z.M. cloned the *Scn2a* promoter sequences and assisted with luciferase assays. N.L. performed PCR genotyping of *Scn8a* transgenic mice and helped with plasmid construction. J.Z. assisted with patch‐clamp recordings. Z.P., X.Y., H.F. and J.L. assisted with ChIP assays. X.M, J.D. and Q.S. assisted with in vitro screening. J.J., C.Y. and H.C. assisted with western blot. Y.Y. assisted with luciferase assays. W.M. provided human epileptic tissues. Z.H., H.S. and J.L. designed the experiments, Z.H. and H.S. wrote the manuscript. Z.H., Y.Y. and Q.S. reviewed the manuscript.

## Supporting information



Supporting Information

Supplemental Table 10

## Data Availability

The bulk RNA‐seq data generated in this study have been deposited in the Zenodo database under accession code https://doi.org/10.5281/zenodo.15687559. Additional supporting data are available in this article and the Supporting Information.
