# Supplementary material for: The DEC2‐SCN2A Axis is Essential for the Anticonvulsant Effects of Cannabidiol by Modulating Neuronal Plasticity
Source: Adv Sci (Weinh). 2025 Jul 11;12(36):e16315. doi: 10.1002/advs.202416315 (PMC12462991; doi:10.1002/advs.202416315)
Supplement: Supplementary file 1 — Supporting Information [file ADVS-12-e16315-s002.docx]

Supporting Information

The DEC2-SCN2A Axis is Essential for the Anticonvulsant Effects of Cannabidiol by Modulating Neuronal Plasticity

Huifang Song, Yifan Wang, Lili Wang, Chang Guo, Shiqi Liu, Yi Rong, Jiawen Tian, Chao Peng, Yuying Shao, Zhixiong Ma, Na Li, Jingliang Zhang, Zijun Peng, Xu Yan, Hangwei Fa, Xinyue Ma, Jie Dong, Jinping Ji, Chen Yang, Haocheng Chen, Jing Liang, Qi Sun*, Yang Yang*, Weining Ma*, Zhuo Huang*

Figures S1-7

Tables S1-9


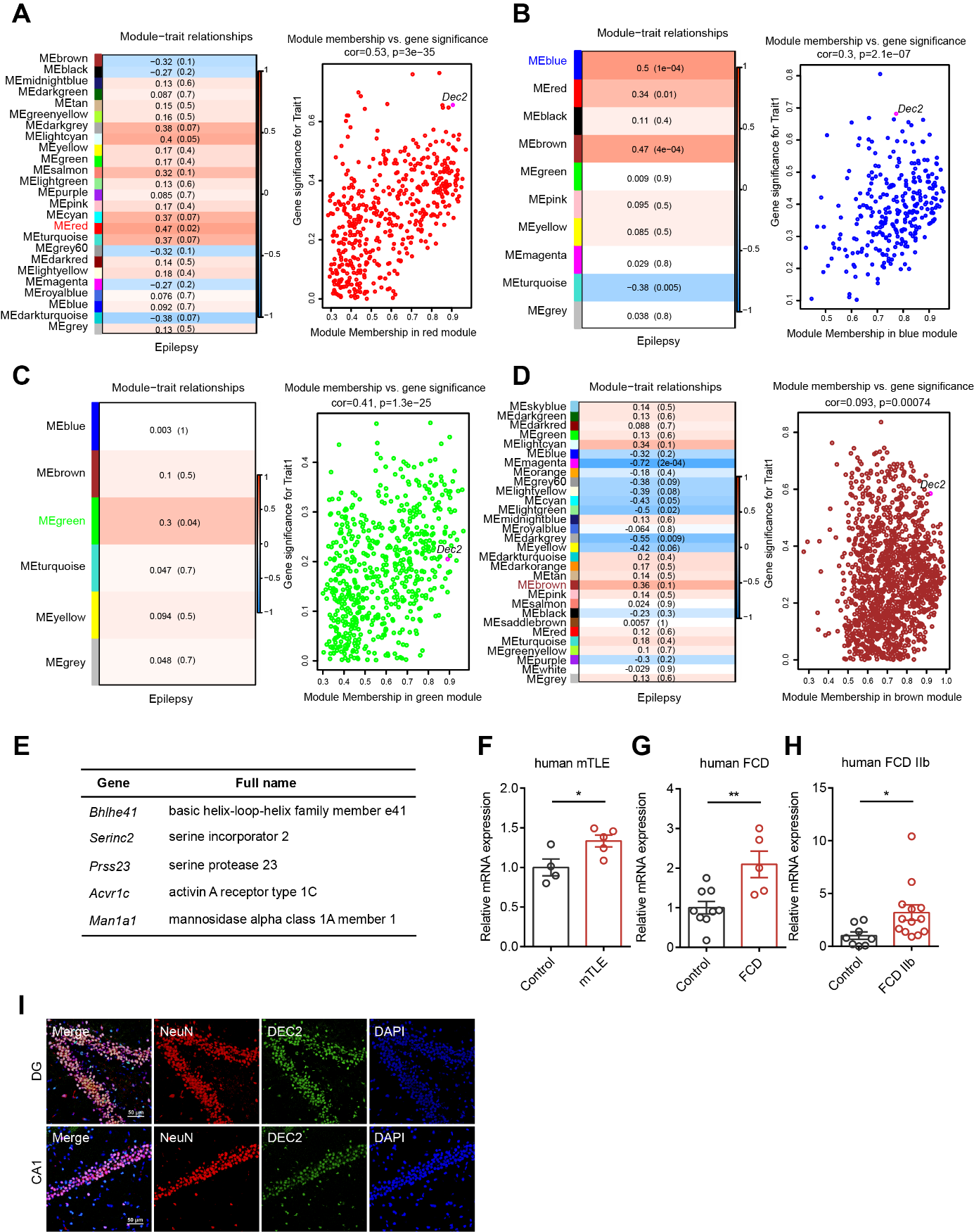


**Figure S1. WGCNA identifies *Dec2* as a highly relevant gene associated with epilepsy.** **(A)** (Left) The module-trait relationships between identified modules (y-axis) and the epilepsy trait (x-axis) in the rat KA model. Each cell in the heatmap displays the correlation coefficient and corresponding *p*-value, with red indicating positive correlation and blue indicating negative correlation. The MEred module shows the strongest positive correlation with epilepsy among all modules. (Right) Scatter plot illustrating the relationship between gene significance for epilepsy (y-axis) and module membership in the MEred module (x-axis). The gene *Dec2* is highlighted in magenta. **(B)** (Left) The module-trait relationships between identified modules (y-axis) and the epilepsy trait (x-axis) in the rat Pilo model. The MEblue module shows the strongest positive correlation with epilepsy among all modules. (Right) Scatter plot illustrating the relationship between gene significance for epilepsy (y-axis) and module membership in the MEblue module (x-axis). The gene *Dec2* is highlighted in magenta. **(C)** (Left) The module-trait relationships between identified modules (y-axis) and the epilepsy trait (x-axis) in the rat kindling model. The MEgreen module shows the strongest positive correlation with epilepsy among all modules. (Right) Scatter plot illustrating the relationship between gene significance for epilepsy (y-axis) and module membership in the MEgreen module (x-axis). The gene *Dec2* is highlighted in magenta. **(D)** (Left) The module-trait relationships between identified modules (y-axis) and the epilepsy trait (x-axis) in the rat SSSE model. The MEbrown module shows the strongest positive correlation with epilepsy among all modules. (Right) Scatter plot illustrating the relationship between gene significance for epilepsy (y-axis) and module membership in the MEbrown module (x-axis). The gene *Dec2* is highlighted in magenta. **(E)** Candidate genes associated with epilepsy identified by cross-model WGCNA analysis. The genes were identified through intersecting the most epilepsy-relevant modules: MEred (KA model), MEblue (Pilo model), MEgreen (kindling model), and MEbrown (SSSE model). **(F-H)** The mRNA levels of *Dec2* in surgical control (n = 4) versus mTLE (n = 5) human specimens (F), control (n = 9) versus FCD (n = 5) human specimens (G), and control (n = 8) versus FCD IIb (n = 13) human specimens (H) by RNA-seq. **p* < 0.05, ***p* < 0.01, unpaired two-tailed Student’s t-test. **(I)** Immunofluorescence staining showing the expression of DEC2 protein in hippocampal DG (upper) and CA1(bottom) area of the mouse brain. Scale bar = 50 μm. Data were represented as mean ± SEM.


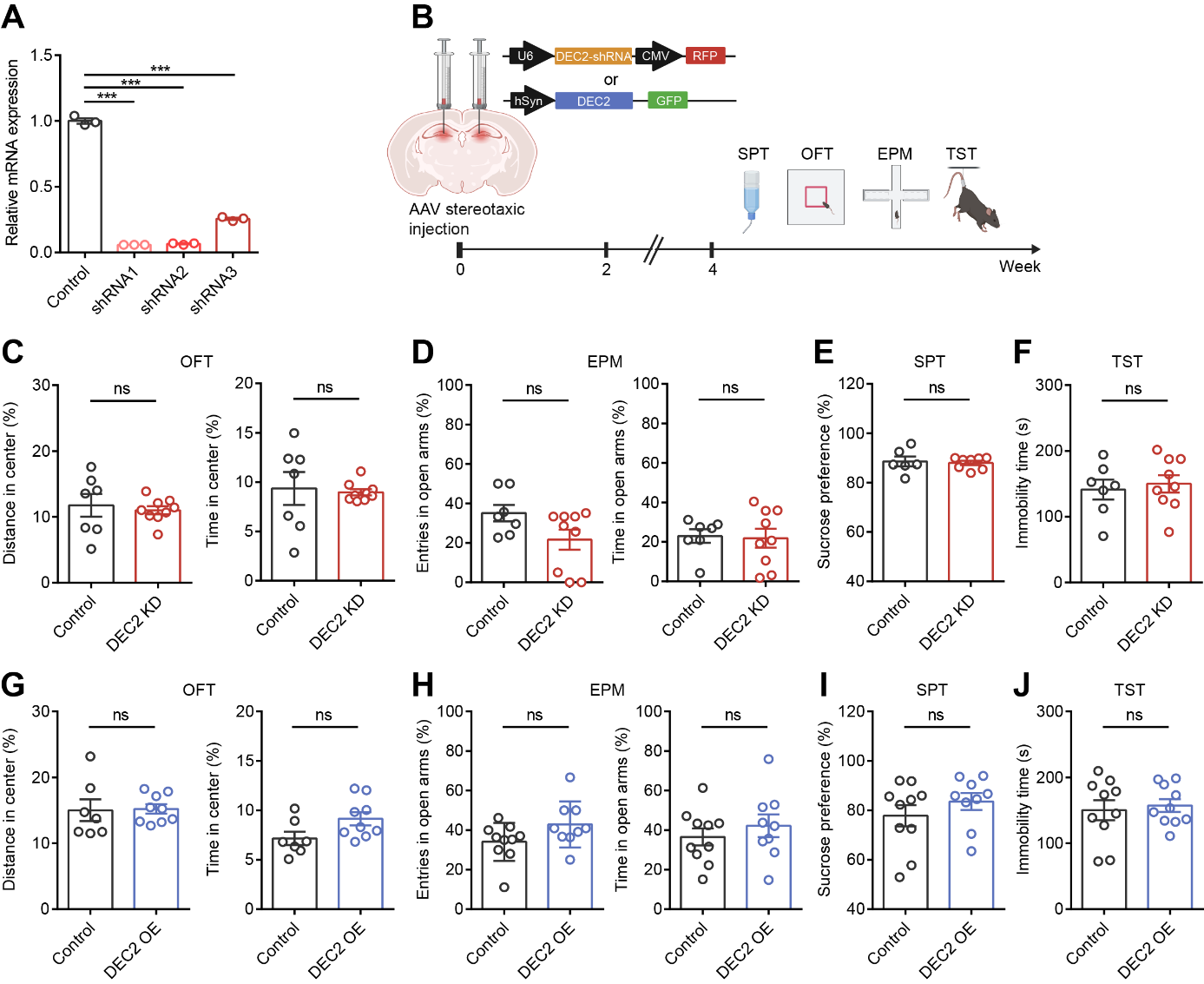


**Figure S2. DEC2 exhibits no effect on locomotor activity, anxiety-related behavior, or depression-related behavior.** **(A)** The mRNA levels of *Dec2* were reduced in shRNA-infected HEK293T cells that overexpressed DEC2 at 72 hours. The levels of mRNA were normalized to that of β-actin. ****p* < 0.001, one-way ANOVA with Bonferroni’s multiple-comparisons test. **(B)** Schematic diagram of the experimental design for viral stereotactic injection and behavioral tests. **(C, D)** Anxiety levels measured by the open field test (C) and the elevated plus maze test (D). n = 7 for control and n = 9 for DEC2 KD, unpaired two-tailed Student’s t-test. **(E, F)** Depression levels measured by the sucrose preference test (E) and the tail suspension test (F). n = 6 for control and n = 8 for DEC2 KD in the SPT, n = 7 for control and n = 9 for DEC2 KD in the TST, unpaired two-tailed Student’s t-test. **(G, H)** Anxiety levels measured by the open field test (G) and the elevated plus maze test (H). n = 7 for control and n = 9 for DEC2 OE in the OFT, n = 10 for control and n = 9 for DEC2 OE in the EPM, unpaired two-tailed Student’s t-test. **(I, J)** Depression levels measured by the sucrose preference test (I) and the tail suspension test (J). n = 10 for control and n = 9 in the SPT, n = 10 per group in the TST, unpaired two-tailed Student’s t-test. Data were represented as mean ± SEM.


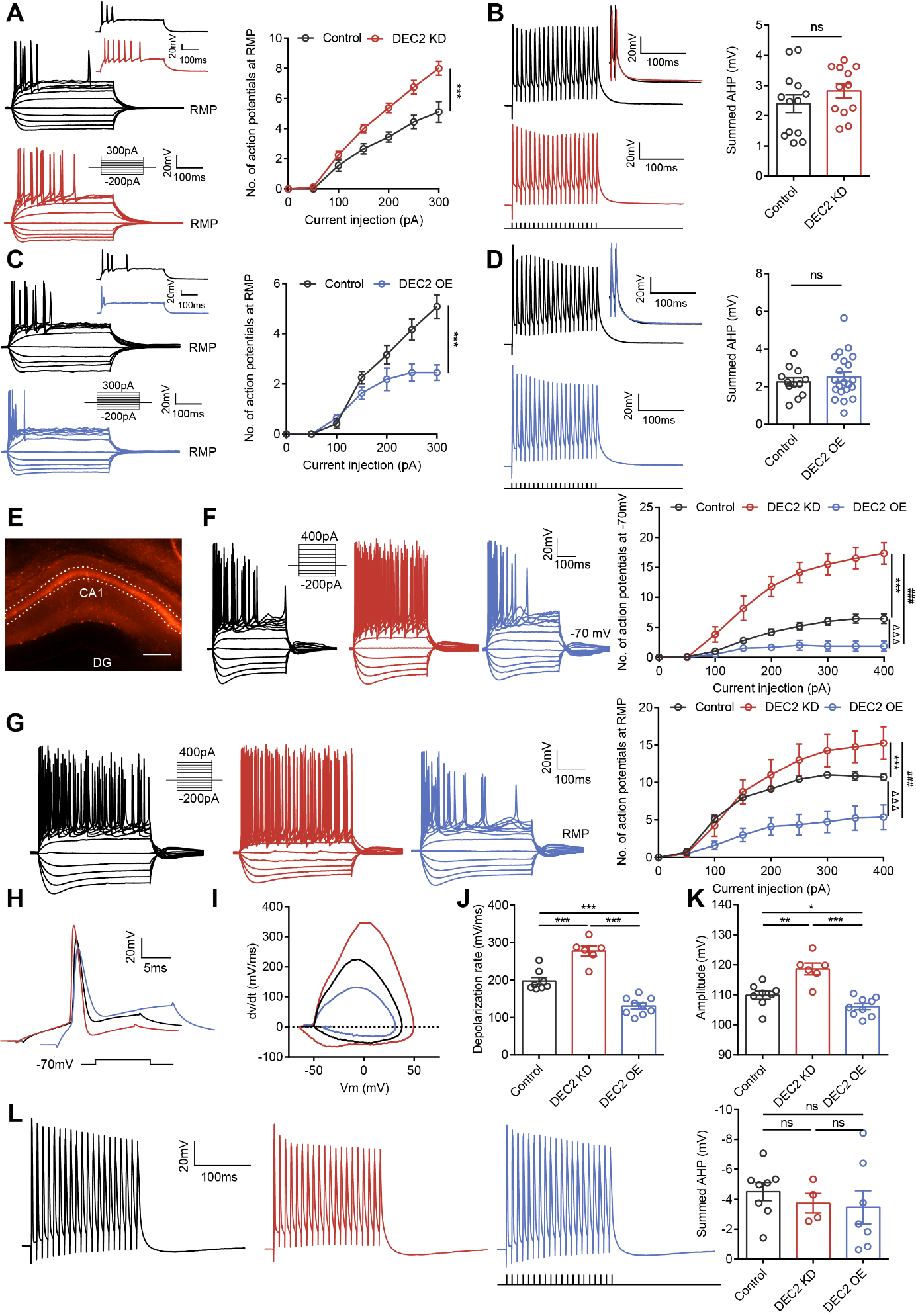


**Figure S3. Knockdown of DEC2 enhances intrinsic neuronal excitability, while overexpression of DEC2 decreases intrinsic neuronal excitability. (A)** Representative current-clamp recordings and the mean number of action potentials generated at RMP in response to depolarizing current pulses obtained from DG neurons of control (black) and DEC2 KD (red) mice. n = 9 for control and n = 8 for DEC2 KD, ****p* < 0.001, two-way ANOVA with Bonferroni’s multiple-comparisons test. **(B)** Summed AHP recorded from DG neurons. 100Hz stimulation was applied to produce the traces. n = 13 for control and n = 12 for DEC2 KD, unpaired two-tailed Student’s t-test. **(C)** Representative current-clamp recordings and the mean number of action potentials generated at RMP in response to depolarizing current pulses obtained from DG neurons of control (black) and DEC2 OE (blue) mice. n = 12 for control and n = 11 for DEC2 OE, ****p* < 0.001, two-way ANOVA with Bonferroni’s multiple-comparisons test. **(D)** Summed AHP recorded from DG neurons. n = 12 for control and n = 21 for DEC2 OE, unpaired two-tailed Student’s t-test. **(E)** Expression of AAV-DEC2 shRNA-RFP at 21 days after infecting the mice CA1 region. Scale bar = 200 μm. (**F)** Representative current-clamp recordings and the mean number of action potentials generated at −70 mV in response to depolarizing current pulses obtained from mice CA1 neurons. n = 9 for control and n = 6 for other groups, control vs DEC2 KD*, ****p* < 0.001, control vs DEC2 OEΔ, ΔΔΔ*p* < 0.001, DEC2 KD vs DEC2 OE#, ###*p* < 0.001, two-way ANOVA with Bonferroni’s multiple-comparisons test. **(G)** Representative current-clamp recordings and the mean number of action potentials generated at RMP in response to depolarizing current pulses obtained from mice CA1 neurons. n = 7 for control, n = 4 for DEC2 KD and n = 8 for DEC2 OE, control vs DEC2 KD*, ****p* < 0.001, control vs DEC2 OEΔ, ΔΔΔ*p* < 0.001, DEC2 KD vs DEC2 OE#, ###*p* < 0.001, two-way ANOVA with Bonferroni’s multiple-comparisons test. **(H-K)** Typical spikes **(H)**, associated phase-plane plots **(I),** depolarization rate **(J)**, and amplitude **(K)** of action potentials obtained from mice CA1 neurons. n = 8 for control, n = 6 for DEC2 KD and n = 9 for DEC2 OE, **p* < 0.05, ***p* < 0.01, ****p* < 0.001, one-way ANOVA with Bonferroni’s multiple-comparisons test. (**L)** Summed AHP recorded from CA1 neurons. n = 8 for control, n = 4 for DEC2 KD and n = 7 for DEC2 OE, one-way ANOVA with Bonferroni’s multiple-comparisons test. Data were represented as mean ± SEM.


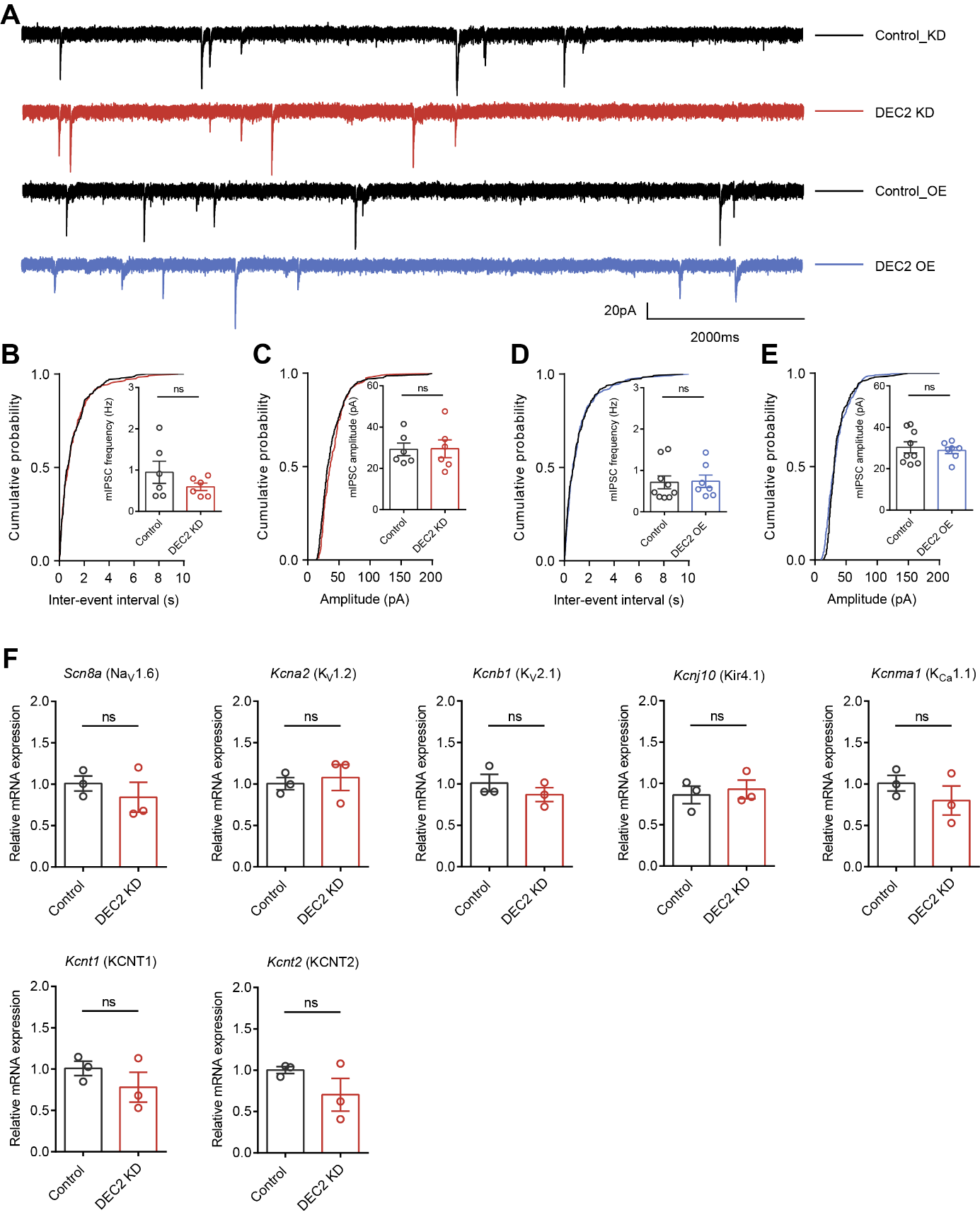


**Figure S4. DEC2 does not alter mIPSCs in hippocampal DG granule neurons but selectively regulates *Scn2a* expression among action potential-related ion channel genes. (A**) Representative mIPSC recording traces obtained from hippocampal DG granule neurons of virus infected mice. **(B)** Cumulative distributions of the mIPSC interevent intervals and quantifications of mIPSC frequency from DG neurons of control and DEC2 KD mice. n = 6, unpaired two-tailed Student’s t-test. **(C)** Cumulative distributions of the mIPSC amplitudes and quantifications of mIPSC amplitudes from control and DEC2 KD mice. n = 6, unpaired two-tailed Student’s t-test. **(D)** Cumulative distributions of the mIPSC interevent intervals and quantifications of mIPSC frequency from control (n = 9) and DEC2 OE (n = 7) mice. Unpaired two-tailed Student’s t-test. **(E)** Cumulative distributions of the mIPSC amplitudes and quantifications of mIPSC amplitudes from control (n = 9) and DEC2 OE (n = 7) mice. Unpaired two-tailed Student’s t-test. **(F)** RT-PCR measuring the mRNA levels of indicated genes. Tissues were obtained from the hippocampus infected with AAV-control and AAV-DEC2 shRNA-RFP virus. The levels of mRNA were normalized to that of β-actin. n = 3, unpaired two-tailed Student’s t-test. Data were represented as mean ± SEM.


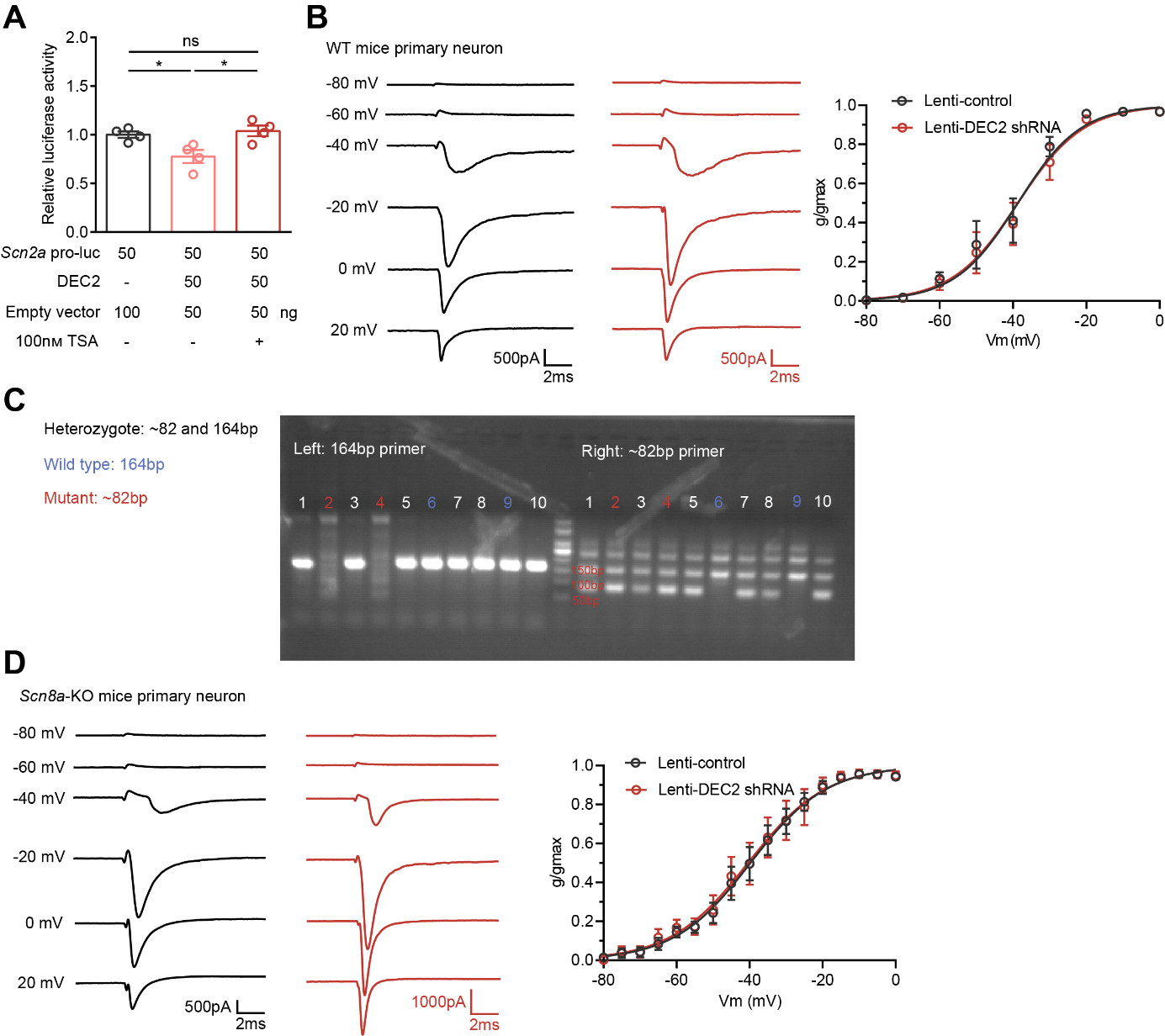


**Figure S5. DEC2 has no significant effect on the activation curve of Na_V_1.2 current. (A)** Effects of HDAC inhibitor on DEC2-mediated transcriptional repression. n = 4, **p* < 0.05, one-way ANOVA with Bonferroni’s multiple-comparisons test. **(B)** Representative whole-cell current recordings on mice primary neurons infected with control (black) and lenti-DEC2 shRNA (red) and voltage-dependence of activation for the Na_V_1.2 channel. n = 7 for control and n = 11 for lenti-DEC2 shRNA infected neurons. Conductance-voltage (*G*–*V*) relationships were fitted to Boltzmann equations. **(C)** Representative genotyping PCR products of offspring mice with different genotypes. The 164 bp band is specific for wild type mice, the ~82 bp band is specific for *Scn8a*-KO mice. The presence of both 164 bp and ~82 bp band indicates a heterozygote. **(D)** Representative whole-cell currents recordings on *Scn8a*-KO mice primary neurons infected with control (black) and lenti-DEC2 shRNA virus (red) and voltage-dependence of activation for the Na_V_1.2 channel. n = 16 for control and n = 10 for lenti-DEC2 shRNA infected neurons. Conductance-voltage (*G*-*V*) relationships were fitted to Boltzmann equations. Data were represented as mean ± SEM.


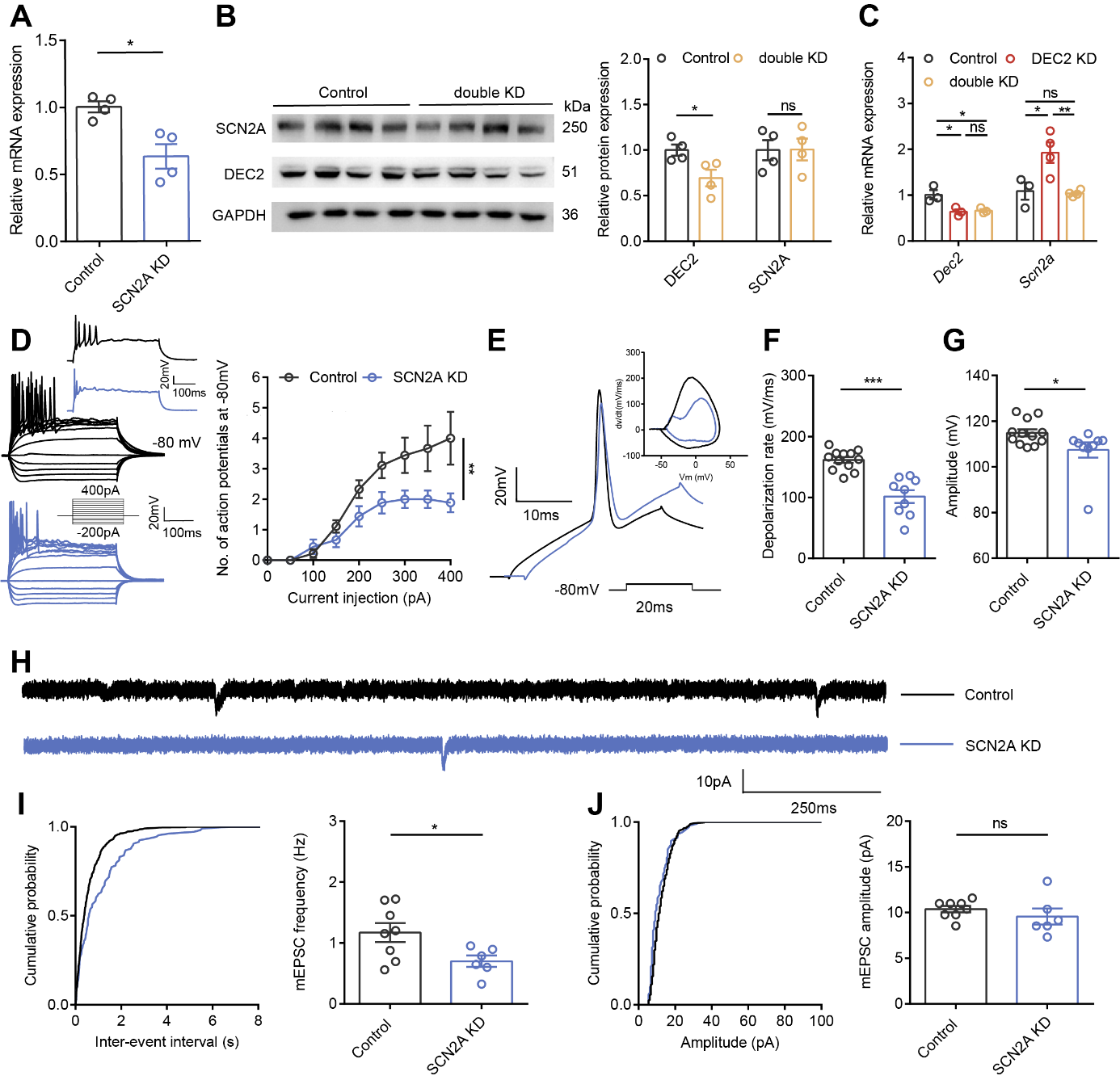


**Figure S6. Loss of SCN2A impairs intrinsic excitability and excitatory synaptic function of hippocampal DG neurons. (A)** The mRNA levels of *Scn2a* in hippocampal DG tissues obtained from mice infected with AAV-control and AAV-SCN2A shRNA for 21 days. The levels of mRNA were normalized to that of β-actin. n = 4, **p* < 0.05, unpaired two-tailed Student’s t-test. **(B, C)** Western blot and RT-PCR showing that infection of AAV-SCN2A shRNA-GFP effectively counteracted the elevation of SCN2A levels induced by DEC2 knockdown in mice hippocampus. n = 4 for western blot and n = 3 to 4 for RT-PCR, **p* < 0.05, ***p* < 0.01, unpaired two-tailed Student’s t-test (B) and one-way ANOVA with Bonferroni’s multiple-comparisons test (C). **(D)** Representative current-clamp recordings and the mean number of action potentials generated in response to depolarizing current pulses obtained from DG neurons of control (black) and SCN2A KD (blue) mice. n = 9 per group, ***p* < 0.01, two-way ANOVA with Bonferroni’s multiple-comparisons test. (**E-G)** Typical spikes and associated phase-plane plots **(E)**, depolarization rate **(F)**, and amplitude **(G)** of action potentials obtained from DG neurons of control and SCN2A KD mice. n = 12 for control and n = 9 for SCN2A KD, **p* < 0.05, ****p* < 0.001, unpaired two-tailed Student’s t-test. **(H)** Representative mEPSC recording traces. (**I)** Cumulative distributions of the mEPSC interevent intervals and quantifications of mEPSC frequency from DG neurons of control (n=8) and SCN2A KD (n = 6) mice. **p* < 0.05, unpaired two-tailed Student’s t-test. **(J)** Cumulative distributions of the mEPSC amplitudes and quantifications of mEPSC amplitudes from DG neurons of control (n = 8) and SCN2A KD (n = 6) mice. Unpaired two-tailed Student’s t-test. Data were represented as mean ± SEM.

**
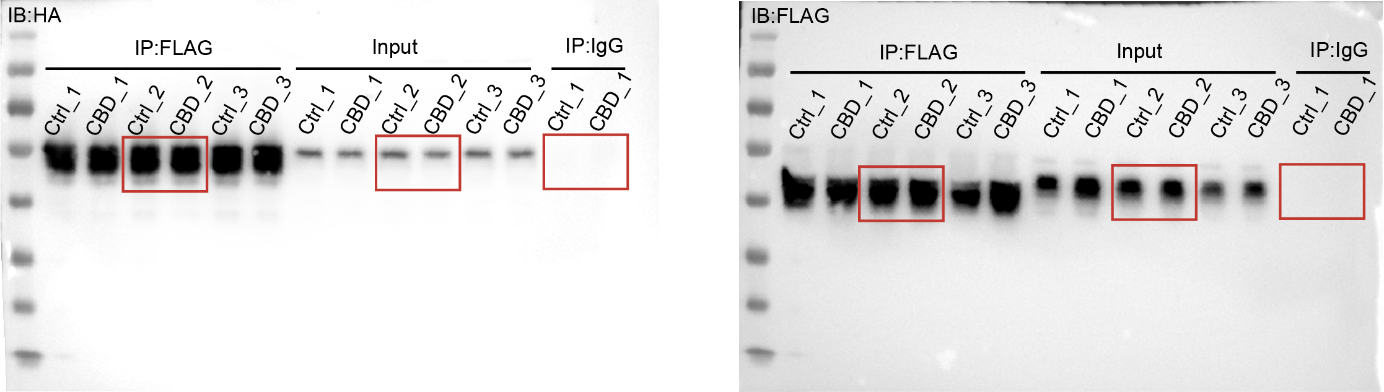
**

**Figure S7. CBD does not affect DEC2-MYOD1 interaction in co-immunoprecipitation assays. Shown are full-length original images corresponding to Figure 7M in the main text (red rectangle indicates cropped region displayed in Figure 7M).** Left panel: Immunoblot (IB) with anti-HA antibody following immunoprecipitation (IP) using anti-FLAG antibody. Right panel: IB with anti-FLAG antibody after IP with anti-FLAG antibody. Controls include input lysate and anti-IgG control. Lanes labeled number represent experimental replicates or different conditions tested. All samples were prepared from HEK293T cell lysates.

**Table S1. Demographic and clinical characteristics of selected patients with mesial TLE**

| Patients | Age | Sex | Duration of disease | Medication history | MRI examination | Type of surgery |
| --- | --- | --- | --- | --- | --- | --- |
| NC1 | 64 | Male | \ | \ | \ |  |
| NC2 | 68 | Male | \ | \ | \ |  |
| NC3 | 17 | Female | \ | \ | \ |  |
| NC4 | 35 | Male | \ | \ | \ |  |
| NC5 | 19 | Male | \ | \ | \ |  |
| NC6 | 22 | Male | \ | \ | \ |  |
| NC7 | 30 | Male | \ | \ | \ |  |
| NC8 | 20 | Male | \ | \ | \ |  |
| SE1 | 32 | Female | 19 | Sodium valproate, Gabapentin | Hippocampal sclerosis | Temporal lobe resection |
| SE2 | 26 | Female | 15 | Sodium valproate, Oxcarbazepine | Right hippocampal atrophy |  |
| SE3 | 28 | Female | 27 | Oxcarbazepine | Left hemisphere atrophy |  |
| SE4 | 35 | Female | 27 | Carbamazepine,  Oxcarbazepine, Clonazepam | Demyelinating lesion |  |
| SE5 | 64 | Female | 30 | Sodium valproate | Focal abnormal signal lesion is observed in the right temporal lobe |  |
| SE6 | 25 | Male | 23 | Trazodone, Carbamazepine | Multiple intracranial hypometabolic areas |  |
| SE7 | 35 | Male | 16 | Valproate, sodium Valproate | Right hippocampal sclerosis |  |
| SE8 | 36 | Female | 14 | Carbamazepine, Valproate | Right hippocampal atrophy, bilateral temporal lobe hypometabolism |  |

**Table S2. Electrophysiological properties of hippocampal DG neurons from control and DEC2 knockdown (KD) mice.**

| Parameters | Control (n = 14) | DEC2 KD (n = 14) |
| --- | --- | --- |
| Resting potential (mV) | −77.71±0.80 | −77.47±0.95 |
| Input resistance (MΩ) | 192.80±15.86 | 205.50±14.26 |
| Sag ratio (Max/SS) | 0.96±0.01 | 0.95±0.01 |
| AP threshold (mV) | −46.44±0.69 | −47.62±0.98 |
| AP depolarization rate (mV/ms) | 132.60±6.44 | 165.20±10.60* |
| AP amplitude (mV) | 114.20±1.11 | 119.70±1.23** |
| AP repolarization rate (mV/ms) | −40.18±2.46 | −51.92±2.87** |
| AP half-width (ms) | 1.77±0.13 | 1.45±0.09* |
| AP AHP (mV) | −5.80±0.72 | −9.25±1.23* |
| Summed AHP (mV) | 2.40±0.29 | 2.82±0.24 |

Data are reported as mean ± SEM for cells of hippocampal DG of mice. **p* < 0.05, ***p* < 0.01, unpaired two-tailed Student’s t-test.

**Table S3. Electrophysiological properties of hippocampal DG neurons from control and DEC2 overexpression (OE) mice.**

| Parameters | Control (n = 18) | DEC2 OE (n = 16) |
| --- | --- | --- |
| Resting potential (mV) | −78.02±0.92 | −77.89±0.50 |
| Sag ratio (Max/SS) | 0.96±0.01 | 0.97±0.01 |
| AP threshold (mV) | −45.95±0.69 | −47.41±0.82 |
| AP depolarization rate (mV/ms) | 151.90±5.43 | 123.90±6.03** |
| AP amplitude (mV) | 114.30±0.78 | 110.40±0.79** |
| AP repolarization rate (mV/ms) | −43.20±1.32 | −37.54±1.67* |
| AP half-width (ms) | 1.61±0.04 | 2.00±0.10*** |
| AP AHP (mV) | −6.09±0.72 | −1.86±1.03** |
| Summed AHP (mV) | 2.42±0.40 | 2.63±0.43 |

Data are reported as mean ± SEM for cells of hippocampal DG of mice. **p* < 0.05, ***p* < 0.01, ****p* < 0.001, unpaired two-tailed Student’s t-test.

**Table S4. Electrophysiological properties of hippocampal CA1 neurons from control, DEC2 knockdown (KD), and DEC2 overexpression (OE) mice.**

| Parameters | Control (n = 8) | DEC2 KD (n = 6) | DEC2 OE (n = 9) |
| --- | --- | --- | --- |
| Resting potential (mV) | −67.44±1.08 | −67.51±1.22 | −68.67±1.52 |
| Input resistance (MΩ) | 157.80±7.42 | 148.20±4.45 | 145.40±10.73 |
| Sag ratio (Max/SS) | 0.76±0.02 | 0.72±0.03 | 0.80±0.04 |
| AP threshold (mV) | −50.60±0.75 | −50.25±1.04 | −51.38±0.61 |
| AP depolarization rate (mV/ms) | 197.60±9.55 | 277.70±13.26*** | 130.50±7.67ΔΔΔ |
| AP amplitude (mV) | 109.80±1.38 | 118.60±1.94** | 106.00±1.04Δ |
| AP repolarization rate (mV/ms) | −44.17±1.43 | −54.03±4.00* | −31.75±1.85ΔΔΔ |
| AP half-width (ms) | 1.81±0.07 | 1.56±0.09* | 2.51±0.10ΔΔΔ |
| AP AHP (mV) | −0.44±0.52 | −9.63±1.70*** | 4.85±1.29ΔΔ |
| Summed AHP (mV) | −4.52±0.60 | −3.74±0.66 | −3.47±1.11 |

Data are reported as mean ± SEM for cells of hippocampal CA1 of mice. Control vs DEC2 KD*, **p* < 0.05, ***p* < 0.01, ****p* < 0.001, Control vs DEC2 OEΔ, Δ*p* < 0.05, ΔΔ*p* < 0.01, ΔΔΔ*p* < 0.001, one-way ANOVA with Bonferroni’s multiple-comparison test.

**Table S5. Gene Ontology (GO) enrichment analysis of upregulated genes in hippocampal tissues from DEC2 knockdown mice.**

| Category | Term | FDR | Gene number | Gene name |
| --- | --- | --- | --- | --- |
| Biological process | Cell adhesion | 1.48E-10 | 49 | ITGB1, PCDHGB7, PCDHGB6, TNC, LAMC2, HAPLN4, STAB1, CCN2, CCN1, NEO1, PCDHGA8, PCDHGA7, PCDHGA6, CXADR, TNFRSF12A, ANXA2, DST, VWF, EPHA8, IZUMO1, OMD, PCDHGA9, COL6A2, COL6A1, CD44, DSC3, PCDH10, SDC3, PCDH15, PBXIP1, CD99L2, THBS2, THBS1, DPP4, ADGRG1, PODXL, PDPN, SPP1, PCDHA3, SUSD5, CADM1, LAMB2, GLYCAM1, PCDH7, L1CAM, COL5A1, KITL, PTK7, PCDHB9 |
|  | Extracellular matrix organization | 4.00E-06 | 23 | VIT, ERO1A, PDGFRA, ELN, COL23A1, OLFML2A, AGT, SMARCA4, COL1A1, ADAMTS15, COL2A1, MMP16, ADAMTSL5, CRISPLD2, ADAMTS1, SPOCK2, COL5A3, MMP17, MMP28, COL4A6, COL4A5, COL9A2, CCN1 |
|  | Negative regulation of cell population proliferation | 5.87E-06 | 34 | PPP1R15A, ITGB1, KANK2, BTG2, CDKN1A, FOXO4, THBS1, PTPRF, CDH5, ING5, ADGRG1, ALOX8, TMEM127, RPS6KA2, PDPN, BDKRB2, TIMP2, E2F7, VDR, DUSP1, CAV1, ZBTB16, LIF, DHCR24, PODN, AGT, ESR2, BMP5, ALDH1A2, CDH13, TAF6, TAX1BP3, NUPR1, ATF5 |
|  | Homophilic cell adhesion via plasma membrane adhesion molecules | 2.06E-05 | 21 | PCDHGA8, PCDHGA7, PCDHGB7, PCDHGA6, PCDHGB6, CADM1, PCDH10, PCDH7, PCDH15, L1CAM, PTPRF, PCDHGA9, CDH5, IGSF21, DCHS2, CDH20, FAT2, CDH13, PCDHA3, PCDHB9, DSC3 |
|  | Modulation of chemical synaptic transmission | 0.001090453 | 17 | ITGB1, UNC13C, DGKE, UNC13A, WNT5A, SLC7A11, L1CAM, ADCY8, CPLX3, PTPRD, AKAP12, GRIN3A, NXPH1, BSN, MET, SHANK3, GRIA4 |
|  | Angiogenesis | 0.001195653 | 24 | MINAR2, SEMA4A, ANGPT2, PLXND1, TNFRSF12A, ANGPT1, ANXA2, CFH, CAV1, RORA, HIF3A, THBS1, MYDGF, ADGRG1, ADGRA2, RHOJ, AMOTL1, CCN2, ANGPTL4, NAA15, MAP3K7, FGFR2, HBEGF, HTATIP2 |
|  | Cell migration | 0.001280128 | 25 | ITGB1, CD151, SDC2, SDC3, PBXIP1, THBS1, PTPRF, CDH5, LIMA1, ADGRG1, PODXL, CDH20, PDPN, CCN2, IGFBP6, PDGFRA, LAMB2, WNT5A, PTK6, L1CAM, COL5A1, PTK7, CDH13, CD44, HBEGF |
|  | Apoptotic process | 0.001929019 | 36 | PPP1R15A, NOTCH2, KANK2, CSRNP1, STEAP3, PLEKHF1, ASAH2, PRUNE2, SLC40A1, HIF3A, THBS1, NTN1, MYDGF, PIDD1, MECOM, RFK, EPB41L3, FADD, CCN1, RNF130, MAP3K7, HTATIP2, PEA15A, MINAR2, AREL1, CADM1, DIO3, NSG1, UNC5D, PLSCR1, TMEM214, KITL, ALDH1A1, COL6A1, BCL2L2, FGFR2 |
|  | Negative regulation of angiogenesis | 0.006180175 | 13 | SEMA4A, ANGPT2, GADD45A, LIF, ATP2B4, FOXO4, THBS2, THBS1, AGT, DCN, MECP2, ADAMTS1, STAB1 |
|  | Rho protein signal transduction | 0.011502587 | 10 | ARHGAP32, ADGRG1, VANGL2, ARHGDIA, RHOJ, PDPN, CDC42EP3, EPS8L1, CDH13, TAX1BP3 |
|  | Neuron differentiation | 0.011582618 | 20 | ITGB1, BRSK1, BTG2, BRSK2, EDN3, WNT5B, MYT1L, WNT5A, FOS, GDPD5, CHRDL1, PTPRD, MECP2, NR4A2, FUT9, ALDH1A2, A830082K12RIK, BAHCC1, MET, WNT4 |
|  | Signal transduction | 0.013134115 | 46 | IL21, PNCK, PDE1C, TENM4, CHRNA4, ARHGAP18, RND2, OLFML2A, FNBP1L, ARHGAP6, DUSP16, PTPRF, TTBK1, GLDN, AKAP12, FAM83H, SOCS3, NRAS, GRK3, PDE11A, SCUBE1, CASKIN1, PDPN, SPP1, FADD, CCN2, SRGAP3, CCN1, SH2B2, WNT4, WNT5B, DUSP1, IZUMO1, WNT5A, PPP2R5A, PRKAB1, ANK1, MAPK8IP1, PTPRD, ARHGAP32, BMP3, OLFM2, RHOJ, IL6ST, PTPN5, CSNK1G2 |
|  | Positive regulation of apoptotic process | 0.013443524 | 26 | PPP1R15A, NOTCH2, ITGB1, STEAP3, UACA, PTPRF, ING5, PIDD1, AIFM2, RPS6KA2, FADD, CCN2, CCN1, TNFRSF12A, GADD45A, DUSP1, ZBTB16, ANXA5, DKKL1, ESR2, PLSCR1, ALDH1A2, ALDH1A1, TAF6, BCL2L2, SLC27A4 |
|  | Cell population proliferation | 0.017963385 | 22 | NOTCH2, ITGB1, RIAN, EDN3, CAV2, CAV1, SPHK1, ZBTB16, WNT5A, LIF, BRCA2, NTN1, AGT, ESR2, MECP2, DLG1, KITL, ALDH1A2, CARM1, MAB21L1, PDPN, FGFR2 |
|  | Nervous system development | 0.023946886 | 22 | SMARCD1, BRSK1, SEMA4A, BRSK2, PHF10, MYT1L, SDC2, NR2F1, FOS, SEMA4G, PTPRF, SMARCA4, PTPRD, NR4A2, CDC20, DLG1, NAV3, FUT9, SCN2A, RXRG, NES, SH2B2 |
|  | Positive regulation of transcription by RNA polymerase II | 0.023946886 | 57 | ZCCHC12, CSRNP1, GMEB2, MYT1L, CD81, CRTC1, SLC40A1, RORA, HIF3A, MYSM1, RPS6KA4, MECP2, MYDGF, MECOM, ZMIZ2, CCN1, MYBL1, HTATIP2, TEAD4, KLF12, ANXA2, WNT5A, TET1, FOS, DCN, PLSCR1, CDH13, ATF5, MET, ATF3, NOTCH2, ARX, FOXO4, HIVEP1, FADD, RXRG, E2F7, S100A10, ZFP677, VDR, AP3D1, NR2F1, LIF, CELA1, MEIS2, ESR2, KLF2, BMP5, SMARCA4, NR4A2, BMP3, CARM1, NFIC, MAFF, FOSB, LPIN3, FGFR2 |
|  | Positive regulation of GTPase activity | 0.023946886 | 12 | ITGB1, DENND1B, RALBP1, CAV2, SGSM2, WNT5A, RAPGEF1, RAPGEF6, ARHGAP6, RGS6, WNT4, S100A10 |
|  | Negative regulation of growth | 0.023946886 | 5 | ING5, ALOX8, MT2, PTK6, MT1 |
|  | Protein O-linked glycosylation | 0.024051167 | 9 | B3GNT9, GALNT15, FUT9, B3GALT2, TET1, ST8SIA6, B3GALT5, TRAK1, TRAK2 |
|  | Liver development | 0.025103645 | 12 | NOTCH2, VWF, CADM1, ALDH1A2, SLCO1B2, COBL, HMGCS2, ACO2, CP, MET, WNT4, SMARCA4 |
|  | Positive regulation of fibroblast proliferation | 0.026022612 | 10 | PDGFRA, NRAS, CCNB1, CDKN1A, ANXA2, SPHK1, WNT5A, FNDC3B, LIF, AGT |
|  | Positive regulation of cell-substrate adhesion | 0.026022612 | 8 | VIT, ITGB1, SPOCK2, SPP1, CCN1, L1CAM, THBS1, FBLN2 |
|  | Post-embryonic development | 0.034258765 | 12 | NR4A2, MECP2, CSRNP1, MECOM, HEG1, SCN9A, EHBP1L1, SLC4A10, CELA1, ATF5, FGFR2, DLK1 |
|  | Positive regulation of cell migration | 0.034258765 | 19 | ITGB1, PDGFRA, SEMA4A, CEMIP, CD151, WNT5B, CAV1, SPHK1, LAMC2, SEMA4G, THBS1, FAM83H, COL1A1, CDH5, PODXL, PDPN, CDH13, CCN1, HBEGF |
|  | Amino acid import across plasma membrane | 0.035841643 | 5 | SLC7A5, SLC7A8, SLC6A13, SLC6A20A, SLC7A1 |
|  | Response to mechanical stimulus | 0.038836563 | 9 | COL1A1, BTG2, ANGPT2, CAV1, COL6A1, TNC, FOSB, MEIS2, THBS1 |
|  | Gamma-aminobutyric acid biosynthetic process | 0.040881308 | 4 | SLC38A1, GAD1, ALDH1A1, GAD2 |

**Table S6. Electrophysiological properties of hippocampal DG neurons from control, DEC2 knockdown (KD), and DEC2 and SCN2A knockdown (double KD) mice.**

| Parameters | Control (n = 9) | DEC2 KD (n = 10) | double KD (n = 12) |
| --- | --- | --- | --- |
| Resting potential (mV) | −75.70±0.93 | −77.95±1.22 | −74.03±1.53 |
| Input resistance (MΩ) | 192.70±11.90 | 214.30±13.56 | 218.90±15.16 |
| Sag ratio (Max/SS) | 0.96±0.01 | 0.95±0.01 | 0.94±0.01 |
| AP threshold (mV) | −47.48±0.88 | −45.62±0.69 | −43.97±0.93 |
| AP depolarization rate (mV/ms) | 147.20±6.69 | 173.80±8.07* | 116.20±7.79### |
| AP amplitude (mV) | 111.70±1.17 | 119.20±0.70*** | 108.00±1.59### |
| AP repolarization rate (mV/ms) | −51.59±2.31 | −61.51±2.18** | −51.87±1.90## |
| AP half-width (ms) | 1.48±0.06 | 1.19±0.04*** | 1.49±0.07## |
| AP AHP (mV) | −7.68±0.27 | −9.68±0.74* | −6.60±0.91# |

Data are reported as mean ± SEM for cells of hippocampal DG of mice. Control vs DEC2 KD*, **p* < 0.05, ***p* < 0.01, ****p* < 0.001, DEC2 KD vs double KD#, #*p* < 0.05, ##*p* < 0.01, ###*p* < 0.001, one-way ANOVA with Bonferroni’s multiple-comparisons test.

**Table S7. Electrophysiological properties of hippocampal DG neurons from control and SCN2A knockdown (KD) mice.**

| Parameters | Control (n = 12) | SCN2A KD (n = 9) |
| --- | --- | --- |
| Input resistance (MΩ) | 200.40±13.48 | 186.90±14.53 |
| Sag ratio (Max/SS) | 0.95±0.01 | 0.96±0.01 |
| AP threshold (mV) | −44.97±0.81 | −45.01±1.00 |
| AP depolarization rate (mV/ms) | 161.40±4.77 | 101.50±10.47*** |
| AP amplitude (mV) | 114.90±1.58 | 107.40±3.32* |

Data are reported as mean ± SEM for cells of hippocampal DG of mice. **p* < 0.05, ***p* < 0.01, ****p* < 0.001, unpaired two-tailed Student’s t-test.

**Table S8. RT-PCR primers**

| Gene | Strand | Sequence |
| --- | --- | --- |
| *Dec2* | F | CCAAAAGGAGCTTGAAGCGAG |
|  | R | ACCGGCGATTTCAGAGAGC |
| *β-actin* | F | GGCTGTATTCCCCTCCATCG |
|  | R | CCAGTTGGTAACAATGCCATGT |
| *Scn2a* | F | ATTTTCGGCTCATTCTTCACACT |
|  | R | GGGCGAGGTATCGGTTTTTGT |
| *Shank3* | F | CGGACCTGCAACAAACGAAG |
|  | R | GCTATGATTGAGGGCGCAGA |
| *Nrxn1* | F | CTTTATGGGCTGTCTCAAAGAGG |
|  | R | CGGGGTCTCAAAAGTTATTGGG |
| *Trak1* | F | CATTACAAGCTAAGAGCGGACA |
|  | R | GATCTGCTCAGTTGTGAGGTC |
| *Slc4a10* | F | GCCACGCTTTCATTACACAGC |
|  | R | CGGACAATAGGAATCCTGTTAGC |
| *Gad2* | F | CATGGTCATCTCAAACCCTGC |
|  | R | CGAGGCGTTCGATTTCTTCA |
| *Gpr37* | F | CCACGAAGAACTGGACAACTC |
|  | R | CAGACCCGTTTTGCGGTAG |
| *Fgfr2* | F | GCCTCTCGAACAGTATTCTCCT |
|  | R | GCCCATCAGGCCCGTATTTAC |
| *Scn8a* | F | GGTCCAAGAATGTGGAGTACA |
|  | R | GCCCTCAGTACCCTGAAAGT |
| *Kcna2* | F | GCACCCACAAGACACCTATGA |
|  | R | GTCTCTGGGAACTGGGCTAAG |
| *Kcnb1* | F | AGAAACACACAGCAATAGCGT |
|  | R | GTACTCCCGTGGAGACTCTTG |
| *Kcnj10* | F | GTCGGTCGCTAAGGTCTATTACA |
|  | R | GGCCGTCTTTCGTGAGGAC |
| *Kcnma1* | F | TCACGGAACTCGCTAAGCC |
|  | R | AATGTGCGTCCCACTGTTTTT |
| *Kcnt1* | F | GGGGCTGCACGAAGTATAACT |
|  | R | TGCCTTTGTAGCTGAGGTAAATG |
| *Kcnt2* | F | CCGGGATTTGCTGCTAGGG |
|  | R | GCGGATTCTTAGACTTGATCGC |
| *Myod1* | F | ATGATGACCCGTGTTTCGACT |
|  | R | CACCGCAGTAGGGAAGTGT |

**Table S9. ChIP and qChIP primers.**

| Gene | Strand | Sequence |
| --- | --- | --- |
| #1 (−133 ~ −32 bp) | F | ACTTGATGCTCAGTGTCCTGTGATT |
|  | R | GCTCCTCGCATAAGAAAGTGCTACA |
| #2 (−383 ~ −106 bp) | F | ACACAAACGAAACAGCAAGCATACA |
|  | R | GGCAATCACAGGACACTGAGCAT |
| #3 (−1491 ~ −1313 bp) | F | TTCTTGATGCCTGTGCCTACCG |
|  | R | CAACATAAGCTGCTGCTGCATAGTT |
| #4 (−1925 ~ −1801 bp) | F | TCTTCACAGTTCCCAGCCCTCTA |
|  | R | AGACCACACCTAACTTCCTGTAAGA |
